# The KRAS-Variant and Cetuximab in HPV-Positive Oropharyngeal Cancer: Results from the NRG/RTOG 1016 Trial

**DOI:** 10.1158/2767-9764.CRC-25-0551

**Published:** 2026-03-31

**Authors:** Joanne B. Weidhaas, Jonathan Harris, Maura L. Gillison, Dukagjin Blakaj, Greg A. Krempl, Kristin Higgins, Jack Phan, Neal E. Dunlap, Shazia T. Mahamood, Jennifer Dorth, Jimmy J. Caudell, Anand B. Desai, Thomas J. Galloway, J. Daniel Pennington, Adam Currey, Jennifer Lathrop, Pedro A. Torres-Saavedra, D. Neil Hayes, Sue S. Yom, Quynh-Thu Le

**Affiliations:** 1 UCLA, Los Angeles, California.; 2 Statistics and Data Management Center, NRG Oncology, Philadelphia, Pennsylvania.; 3 American College of Radiology, Philadelphia, Pennsylvania.; 4 The University of Texas MD Anderson Cancer Center, Houston, Texas.; 5 Ohio State University Comprehensive Cancer Center, Columbus, Ohio.; 6 University of Oklahoma College of Medicine, Oklahoma City, Oklahoma.; 7 Emory University Hospital, Winship Cancer Institute, Atlanta, Georgia.; 8 The James Graham Brown Cancer Center at University of Louisville, Louisville, Kentucky.; 9 Allan Blair Cancer Centre, Regina, Saskatchewan, Canada.; 10 University Hospitals Seidman Cancer Center, Cleveland, Ohio.; 11 Moffitt Cancer Center, Tampa, Florida.; 12 Summa Health System – Akron Campus, Akron, Ohio.; 13 Fox Chase Cancer Center, Philadelphia, Pennsylvania.; 14 NCORP, Southeast Clinical Oncology Research Consortium, Winston-Salem, North Carolina.; 15 Radiation Oncology Associates, Richmond, Virginia.; 16 Zablocki VA Medical Center, Medical College of Wisconsin, Milwaukee, Wisconsin.; 17 University of Tennessee Health Science Center, Memphis, Tennessee.; 18 University of California, San Francisco, San Francisco, California.; 19 Stanford Cancer Institute Palo Alto, Stanford, California.

## Abstract

**Purpose:**

NRG/RTOG 1016 was a phase III noninferiority trial comparing IMRT + cisplatin versus IMRT + cetuximab for human papillomavirus–positive oropharyngeal squamous cell cancer (HPV+ OPSCC). A germline mutation (the *KRAS-*variant) previously identified patients with improved outcomes to radiation + cetuximab + cisplatin; thus, we investigated whether there may be similar benefits for IMRT + cetuximab.

**Patients and Methods:**

562 patients were tested, with 16% positive for the *KRAS-*variant. Clinical endpoints were per NRG/RTOG 1016, and hazard ratios (HR) were estimated by Cox models. Negative binomial regression modeled treatment-related acute and late grade 3 to 5 adverse events (AE). All models included *KRAS*, assigned treatment, their interaction, and other prognostic variables tested at two-sided 0.05.

**Results:**

There was no association between the *KRAS-*variant and clinical outcomes. There were higher local–regional failure rates (LRF) in nonvariant patients treated with cetuximab [HR = 1.83 (1.19–2.82)] versus *KRAS-*variant patients [HR (95% CI), 0.90 (0.34–2.41)]. Grade 3 to 4 acute mucositis was higher in nonvariant patients treated with cetuximab [OR = 1.41 (95% CI, 0.98–2.03)] and lower in *KRAS-*variant patients [OR = 0.60 (95% CI, 0.25–1.41)]. However, the mean late toxicity ratio was lower in nonvariant patients [0.55 (95% CI, 0.35–0.87)] versus *KRAS-*variant patients [1.62 (95% CI, 0.57–4.62)]. *KRAS* by treatment interaction for acute and late AEs was not significant (*P* = 0.0717 and 0.0659, respectively).

**Conclusions:**

Possible lower LRF and less acute toxicity with cetuximab versus cisplatin for *KRAS-*variant patients seem to be offset by potentially increased late toxicity. Further evaluation of this class of biomarkers is warranted.

**Significance:**

Germline microRNA-disrupting biomarkers are functional biomarkers being studied to help guide treatment personalization in cancer. The work in this study is a hypothesis-generating evaluation of the *KRAS-*variant as a potential biomarker to help predict outcomes and toxicity in patients with head and neck cancer treated with radiation and cetuximab. Findings from this work suggest that these biomarkers may have the potential to differentiate patients at risk of acute versus late toxicity, supporting further study of these biomarkers in radiation studies.

## Introduction

There has been a significant effort to improve the local control of tumors treated with radiation, without causing additional toxicity. This has been especially important, yet challenging, in the treatment of head and neck cancer (HNSCC) due to the nature of the areas being treated. The addition of platinum chemotherapy to radiotherapy in HNSCC has been shown to significantly improve survival, however, with additional acute (1) and late toxicity (2). Early efforts to add cetuximab, an antibody targeting the epidermal growth factor receptor (EGFR), to radiotherapy in HNSCC were promising as it was shown to improve survival without an increase in toxicity over radiotherapy alone (3, 4). Based on these data, but without a direct comparison, these two regimens, radiation plus cisplatin, or radiation plus cetuximab, were proposed to have similar efficacy in HNSCC (5). However, the actual risks and benefits of cisplatin versus cetuximab with radiation were unknown.

A significant advance in HNSCC was the identification of a subgroup of patients with HNSCC who were younger and had improved survival—those with oropharyngeal squamous cell carcinomas that were associated with human papillomavirus (HPV) infection (p16-positive; ref. 6). Because of their improved prognosis and younger age, there has been strong interest in de-escalating therapy with the goal of decreasing late toxicity for these patients. NRG/RTOG 1016 was a randomized, noninferiority, multicenter trial for patients with locoregionally advanced p16-positive oropharyngeal cancer (HPV+ OPSCC) to test the hypothesis that treatment with radiation plus cetuximab would result in noninferior survival outcomes with less late toxicity for these patients compared with radiation plus cisplatin (7).

Unfortunately, the results from NRG/RTOG 1016, as well as a similar trial in low-risk HPV+ OPSCC (De-ESCALate HPV), and the TROG 12.01 trial were all negative (7–9). In fact, all trials reported worse cancer outcomes in the cetuximab-treated arms, with worse 2-year overall survival (OS) and 2-year recurrence in low-risk HPV+ OPSCC (De-ESCALaTE HPV; ref. 8), worse progression-free survival (PFS) and locoregional failure (LRF) for all-risk HPV+ OPSCC (RTOG 1016; ref. 7), and worse failure-free survival without improvement in symptom burden or toxicity in low-risk HPV+ OPSCC (TROG 12.01; ref. 9). Interestingly, NRG/RTOG 1016 reported a decrease in acute toxicity and similar late toxicity for cetuximab-treated versus cisplatin-treated patients, and De-ESCALate and TROG found no differences in acute or late toxicity.

A germline microRNA-disrupting variant in the regulatory region of *KRAS*, referred to as the *KRAS-*variant, has previously been shown to predict a positive response to cetuximab in retrospective analyses of prospective clinical trials for several cancers, including colon, lung [non–small cell lung cancer (NSCLC)], and HNSCC (10–12). In HNSCC, the *KRAS-*variant was evaluated in the RTOG 0522 trial, which compared radiation plus cisplatin with or without the addition of cetuximab. The *KRAS-*variant was found in 17% of RTOG 0522 patients, and these patients were found to benefit significantly from the addition of cetuximab to radiotherapy and cisplatin chemotherapy, with improved PFS for 1 year and OS for 2 years after treatment, which seemed to be most impactful for HPV+ patients. The positive response was hypothesized to be due to the immune-stimulating effects of cetuximab (13–15), as this study also demonstrated that *KRAS*-variant patients had significantly elevated TGF-B1 compared with nonvariant patients, and thus *KRAS*-variant patients were likely immunosuppressed (16). *KRAS*-variant patients were found to have increased grades 3 and 4 mucositis with radiation plus cisplatin, which was not worsened by the addition of cetuximab (47.4% vs. 50%). In contrast, nonvariant patients had lower grades 3 and 4 mucositis with radiation and cisplatin, which was significantly increased with the addition of cetuximab (37.9% vs. 50.6%, *P* = 0.02). Acute and late toxicity were not separated as distinct endpoints in this analysis.

Because *KRAS*-variant patients seemed to respond to cetuximab and radiation (in combination with cisplatin) and seemed to have increased radiosensitivity to radiation plus cisplatin that was not worsened by the addition of cetuximab, we evaluated *KRAS-*variant patients in NRG/RTOG 1016 to see whether there were similar associations with the endpoints of response or toxicity in this study of radiation plus cisplatin versus radiation plus cetuximab.

## Patients and Methods

### Germline *KRAS-*variant detection in DNA

DNA from 562 samples was extracted from plasma, buffy coat, or whole blood and was tested for the *KRAS-*variant using a Taqman-based assay and run in duplicate with appropriate positive and negative controls at MiraDx, a California State Clinical Laboratory Improvement Amendments–certified and College of American Pathologists approved laboratory. A sample was considered positive if it was found to be T/G or G/G, thus heterozygous or homozygous, and wild-type (nonvariant) if T/T.

### Statistical methods

Per NRG/RTOG 1016, outcomes were defined as the following: an event for OS is death due to any cause; an event for PFS is local, regional, or distant progression or death due to any cause; an event for LRF is local or regional progression, salvage surgery of the primary tumor with tumor present/unknown, salvage neck dissection with tumor present/unknown >20 weeks after the end of radiotherapy, death due to study cancer without documented progression, or death due to unknown causes without documented progression; distant metastasis (DM) and death from other causes were considered competing risks. LRF and death were considered competing risks for the DM endpoint. A patient could not be considered an event for both LRF and DM endpoints. All endpoints were measured from the date of randomization. OS and PFS and number of grade 3 to 5 adverse events (AE) are protocol-specified analyses, but LRF, DM, and AE rates for specific terms are unplanned. SAS (RRID: SCR_008567) was used for statistical analysis.

Median follow-up was estimated by the reverse Kaplan–Meier method. OS and PFS rates were estimated by the Kaplan–Meier method, and LRF and DM rates by the cumulative incidence method. Hazard ratios (HR) for OS and PFS were estimated by Cox proportional hazards models, and for LRF and DM by cause-specific Cox models. Models were compared by Bayesian information criterion (BIC); lower BIC indicates better fit. The Cox model assumptions were assessed using diagnostics based on scaled Schoenfeld residuals and cumulative martingale residuals. Cox models to assess the prognostic role of the *KRAS-*variant were stratified by assigned treatment; the *KRAS* effect was tested at one-sided 0.025 for OS and one-sided 0.05 for PFS, LRF, and DM.

To assess the predictive role of the *KRAS-*variant, Cox models with *KRAS*, assigned treatment, and their interaction were used; *KRAS* by treatment interaction effect was tested at two-sided 0.05 for all endpoints. Three sets of multivariable models were performed for each endpoint: base multivariable model without adjustment for additional covariates, full multivariable model adjusted for all covariates, and reduced multivariable model with minimum BIC of all models evaluated. In addition to *KRAS* and assigned treatment, the following variables were considered for inclusion in the models: age, gender, Zubrod performance status, smoking history measured in pack-years, T stage, N stage, and risk group per NRG/RTOG 0129.

Rate ratios were estimated using negative binomial regression modeling the number of treatment-related (definitely, probably, or possibly related to treatment) acute (≤180 from end of treatment) and late (>180 from end of treatment) grade 3 to 5 AEs. Common Terminology Criteria for Adverse Events v. 4 was used for toxicity scoring. Negative binomial regression models included *KRAS*, assigned treatment, and their interaction; the *KRAS* X treatment interaction effect was tested at two-sided 0.05. Odds ratios (OR) were estimated using logistic regression modeling the probability of specific treatment-related grade 3 to 4 AE terms. Logistic regression models included *KRAS*, assigned treatment, and their interaction; the *KRAS* X treatment interaction effect was tested at two-sided 0.05. Grade 3 to 4 treatment-related AE rates over time are estimated in the subgroups defined by *KRAS* and assigned treatment at the following time points: during treatment; 1 month after treatment (window: −14 to +28 days); 3 months after treatment (window: −28 to +42 days); 6 months after treatment (window: −42 to +56 days); and 1 to 5 years after treatment (window: ±91 days). The analysis population for OS, PFS, LRF, and DM is randomized and eligible patients who have *KRAS* data. The analysis population for AEs is randomized and eligible patients who started protocol treatment and have *KRAS* data.

Model assessment for all outcome endpoints did not suggest serious violations of the Cox model assumptions.

### NRG/RTOG 1016 study details, patient inclusion, and exclusion

In NRG/RTOG 1016, patients were stratified by T stage (T1–2 vs. T3–4), N stage (N0–2a vs. N2b–3), Zubrod performance status (0 vs. 1), and smoking history (≤10 pack-years vs. >10 pack-years) and randomized (1:1) to IMRT + cisplatin or IMRT + cetuximab.

A CONSORT flow diagram is shown in Supplementary Fig. S1. Of 987 patients, 849 (86%) were randomized, of whom 805 (94.8%) were included in the analysis of the primary endpoint. Of these 805, 733 (91.1%) consented to blood collection. Of these 733, 562 (76.7%) had sample and data available for *KRAS* and are included in this analysis. The representativeness of the study participants is addressed in Supplementary Table S13.

## Results

### Prevalence and patient and tumor characteristics

Patient and tumor characteristics were largely similar for patients included in and excluded from analysis (Supplementary Table S1). However, outcomes for patients included in the analysis tended to be worse relative to patients excluded: HRs were 1.43 [95% confidence interval (CI), 1.04–1.96] for OS, 1.22 (95% CI, 0.93–1.60) for PFS, 1.27 (95% CI, 0.87–1.86) for LRF, and 0.83 (95% CI, 0.52–1.33) for DM.

The *KRAS-*variant was found in 16% of patients, which was close to the projected prevalence (17%) used in the design stage of the study. Patient and tumor characteristics were similar for patients with and without the *KRAS-*variant and across the treatment arms (Supplementary Table S2). There was a similar percentage of intermediate-risk patients in the *KRAS-*variant (33.7%) versus nonvariant (29.1%) subgroups. The median follow-up was 8.6 years for the entire cohort, 8.3 years for *KRAS*-variant patients, and 8.7 years for nonvariant patients.

### Outcomes

The *KRAS-*variant was not a prognostic marker for OS, PFS, LRF, or DM (Supplementary Tables S3–S6; Supplementary Figs. S2–S5), in agreement with previous findings (16). The *KRAS-*variant was also not a predictive biomarker for OS or PFS, with HRs for treatment effects between variant and nonvariant patients being similar and in the same direction. In the reduced model for OS, the HRs for treatment effect (IMRT + cetuximab/IMRT + cisplatin) were 1.46 (95% CI, 0.65–3.32) for the *KRAS*-variant subgroup and 1.20 (95% CI, 0.86–1.67) for the nonvariant subgroup (*KRAS* by treatment interaction *P* = 0.6540; Supplementary Table S7; Supplementary Fig. S6). For PFS, in the reduced model, the HRs for treatment effect (IMRT + cetuximab/IMRT + cisplatin) were 1.24 (95% CI, 0.61–2.54) in the *KRAS*-variant subgroup and 1.40 (95% CI, 1.03–1.90) in the nonvariant subgroup (*KRAS* by treatment interaction *P* = 0.7700; Supplementary Table S8; Supplementary Fig. S7).

**Figure 1.**
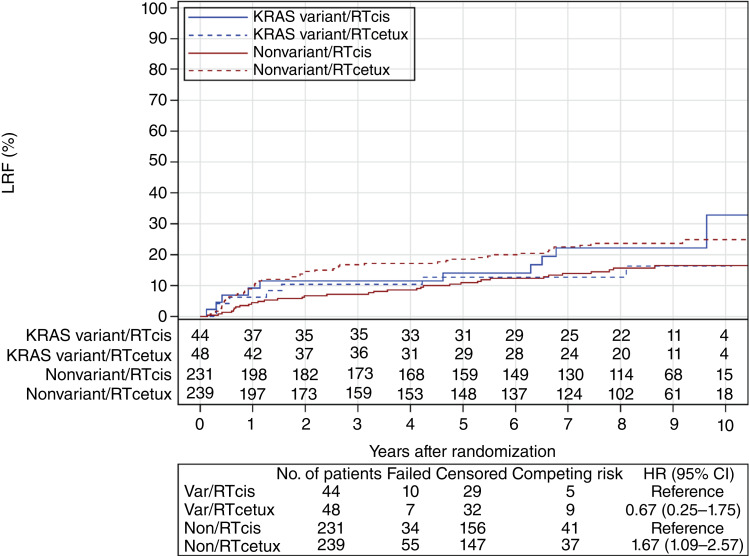
LRF by *KRAS* and assigned treatment. Rates of local failure based on treatment and *KRAS-*variant status, with *KRAS-*variant patients being depicted by blue lines and nonvariant patients by red lines and dashed lines representing cetuximab-treated patients.

**Figure 2.**
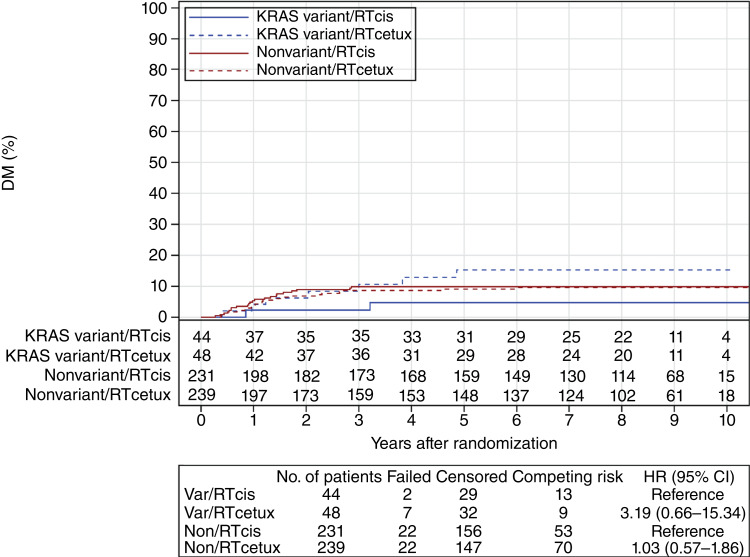
DM by *KRAS* and assigned treatment. Distant failure rates based on treatment and *KRAS-*variant status, with *KRAS-*variant patients being depicted by blue lines and nonvariant patients by red lines and dashed lines representing cetuximab-treated patients.

For LRF and DM, although the *KRAS-*variant was again not predictive, the HRs for treatment effects were in an opposite direction comparing variant and nonvariant patients. For LRF in the base model, the HRs for treatment effect (IMRT + cetuximab/IMRT + cisplatin) were 0.67 (95% CI, 0.25–1.75) for the *KRAS*-variant subgroup versus 1.67 (95% CI, 1.09–2.57) for the nonvariant subgroup (Fig. 1). In the reduced model, the HRs for treatment effect were 0.90 (95% CI, 0.34–2.41) in the *KRAS*-variant subgroup and 1.83 (95% CI, 1.19–2.82) in the nonvariant subgroup (*KRAS* by treatment interaction *P* = 0.1933; Supplementary Table S9). For DM, in the base model, the HRs for treatment effect (IMRT + cetuximab/IMRT + cisplatin) were 3.19 (95% CI, 0.66–15.34) in the *KRAS*-variant subgroup and 1.03 (95% CI, 0.57–1.86) in the nonvariant subgroup (Fig. 2). In the reduced model, the HRs for treatment effect are 3.04 (95% CI, 0.63–14.66) in the *KRAS*-variant subgroup and 1.05 (95% CI, 0.58–1.90) in the nonvariant subgroup (*KRAS* by treatment interaction *P* = 0.2160, Supplementary Table S10).

### Acute toxicity

The mean [standard deviation (SD)] number of acute treatment-related grade 3 to 5 AEs was 3.70 (2.84) for IMRT + cisplatin–treated *KRAS*-variant patients, 1.98 (2.37) for IMRT + cetuximab–treated *KRAS*-variant patients, 3.15 (2.95) for IMRT + cisplatin–treated nonvariant patients, and 2.51 (2.40) for IMRT + cetuximab–treated nonvariant patients (Table 1). The mean acute toxicity ratio comparing IMRT + cetuximab/IMRT + cisplatin was 0.53 (95% CI, 0.36–0.80) in the *KRAS*-variant subgroup and 0.80 (95% CI, 0.67–0.95) in the nonvariant subgroup. This means that the *KRAS*-variant subgroup of patients treated with IMRT + cetuximab had, on average, 47% fewer acute toxicities compared with *KRAS-*variant patients treated with IMRT + cisplatin. The nonvariant subgroup of patients treated with IMRT + cetuximab exhibited, on average, 20% fewer acute toxicities compared with those treated with IMRT + cisplatin. However, the interaction of *KRAS* and treatment was not significant (*P* = 0.0703).

**Table 1.** Number of grade 3–5 treatment-related^a^ acute^b^ AEs by *KRAS* and assigned treatment.

*KRAS*	Assigned treatment	Patients	Mean (SD)	Rate ratio (95% CI)
*KRAS* variant	IMRT + cisplatin	44	3.70 (2.84)	Reference
​	IMRT + cetuximab	48	1.98 (2.37)	0.53 (0.36–0.80)
Nonvariant	IMRT + cisplatin	230	3.15 (2.95)	Reference
​	IMRT + cetuximab	237	2.51 (2.40)	0.80 (0.67–0.95)
Total	​	559	​	Interaction *P* = 0.0703

Rate ratios estimated from negative binomial regression model with covariates KRAS (KRAS variant vs. nonvariant), treatment (IMRT + cetuximab vs. IMRT + cisplatin), and the interaction of KRAS and treatment.

a Definitely, probably, or possibly related to protocol treatment.

b ≤180 days from end of treatment.

Scaled Pearson *χ*^2^ (value/degrees of freedom) = 0.9754.

Treatment-related grade 3 to 4 acute mucositis, within the *KRAS*-variant subgroup, was reported in 18 of 44 (40.9%) patients treated with IMRT + cisplatin and in 14 of 48 (29.2%) treated with IMRT + cetuximab. Within the nonvariant subgroup, treatment-related grade 3 to 4 acute mucositis was reported in 94 of 230 (40.9%) patients treated with IMRT + cisplatin and in 117 of 237 (49.4%) treated with IMRT + cetuximab (Table 2). The OR comparing IMRT + cetuximab/IMRT + cisplatin was 0.60 (95% CI, 0.25–1.41) in the *KRAS*-variant subgroup and 1.41 (95% CI, 0.98–2.03) in the nonvariant subgroup. Again, the interaction of *KRAS* and treatment was not significant (*P* = 0.0717). Although not statistically significant, the finding of worse mucositis with the addition of cetuximab to treatment for nonvariant patients, but not for *KRAS-*variant patients, is consistent with prior findings (16).

**Table 2.** Grade 3–4 treatment-related^a^ acute^b^ mucositis^c^ by *KRAS* and assigned treatment.

*KRAS*	Assigned treatment	Patients	Events	OR (95% CI)
*KRAS* variant	IMRT + cisplatin	44	18 (40.9%)	Reference
​	IMRT + cetuximab	48	14 (29.2%)	0.60 (0.25–1.41)
Nonvariant	IMRT + cisplatin	230	94 (40.9%)	Reference
​	IMRT + cetuximab	237	117 (49.4%)	1.41 (0.98–2.03)
Total	​	559	243 (43.5%)	Interaction *P* = 0.0717

ORs estimated from logistic regression model with covariates KRAS (KRAS variant vs. nonvariant), treatment (IMRT + cetuximab vs. IMRT + cisplatin), and the interaction of KRAS and treatment.

a Definitely, probably, or possibly related to protocol treatment.

b ≤180 days from end of treatment.

c Common Terminology Criteria for Adverse Events version 4 term: mucositis oral.

There were no significant differences between *KRAS-*variant patients versus nonvariant patients for skin reaction inside or outside of the portal (Supplementary Tables S11 and S12).

### Late toxicity

The mean (SD) number of late treatment-related grade 3 to 5 AEs was 0.27 (0.55) for IMRT + cisplatin–treated *KRAS*-variant patients, 0.43 (0.83) for IMRT + cetuximab–treated *KRAS*-variant patients, 0.49 (1.18) for IMRT + cisplatin–treated nonvariant patients, and 0.27 (0.70) for IMRT + cetuximab–treated nonvariant patients (Table 3). The mean late toxicity ratio comparing IMRT + cetuximab/IMRT + cisplatin was 1.62 (95% CI, 0.57–4.62) in the *KRAS*-variant subgroup and 0.55 (95% CI, 0.35–0.87) in the nonvariant subgroup. This means that the *KRAS*-variant subgroup of patients treated with IMRT + cetuximab exhibited, on average, 62% more late toxicities compared with those treated with IMRT + cisplatin. The nonvariant subgroup of patients treated with IMRT + cetuximab exhibited, on average, 45% fewer late toxicities compared with those treated with IMRT + cisplatin. The interaction of *KRAS* and treatment was again not significant (*P* = 0.0659).

**Table 3.** Number of grade 3–5 treatment-related^a^ late^b^ AEs by *KRAS* and assigned treatment.

*KRAS*	Assigned treatment	Patients	Mean (SD)	Rate ratio (95% CI)
*KRAS* variant	IMRT + cisplatin	41	0.27 (0.55)	Reference
​	IMRT + cetuximab	46	0.43 (0.83)	1.62 (0.57–4.62)
Nonvariant	IMRT + cisplatin	221	0.49 (1.18)	Reference
​	IMRT + cetuximab	225	0.27 (0.70)	0.55 (0.35–0.87)
Total	​	533	​	Interaction *P* = 0.0659

Rate ratios estimated from negative binomial regression model with covariates KRAS (KRAS variant vs. nonvariant), treatment (IMRT + cetuximab vs. IMRT + cisplatin), and the interaction of KRAS and treatment.

a Definitely, probably, or possibly related to protocol treatment.

b >180 days from end of treatment.

Scaled Pearson *χ*^2^ (value/degrees of freedom) = 0.9778.

**Figure 3.**
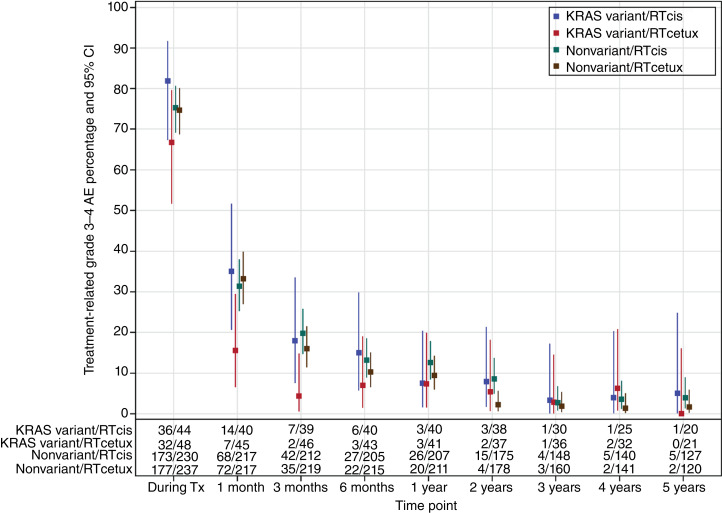
Grade 3 to 4 treatment-related AEs over time by *KRAS* and assigned treatment. Rates of treatment-related AEs with 95% CI by treatment and *KRAS-*variant status, with *KRAS-*variant patients being depicted by blue (cisplatin-treated) and red (cetuximab-treated) and nonvariant patients depicted by green (cisplatin-treated) and brown (cetuximab-treated).

Grade 3 to 4 treatment-related AE rates over time are shown in Fig. 3, allowing the view of toxicity across both subgroups (*KRAS-*variant vs. nonvariant) and all treatment approaches (IMRT + cisplatin vs. IMRT + cetuximab). Visually, this seems to show lower acute toxicity for *KRAS-*variant patients treated with cetuximab compared with *KRAS-*variant patients treated with cisplatin, and nonvariant patients treated with cetuximab or cisplatin through 6 months, but then loss of these differences by 1 year.

## Discussion

In this study, we tested the hypothesis that there would be an association of the *KRAS-*variant, the first germline microRNA-associated mutation discovered in cancer, and HPV+ OPSCC patient outcome with treatment with radiation plus cetuximab versus radiation plus cisplatin. This study did not support the hypothesis that the *KRAS-*variant is a predictive biomarker of improved outcome in patients with HPV+ OPSCC treated with IMRT + cetuximab. However, there was a nonsignificant trend for a lower rate of LRF with cetuximab for the *KRAS-*variant subgroup of patients, in contrast to what is seen in nonvariant patients who exhibit a significantly higher risk of LRF. In addition, acute toxicity for *KRAS-*variant patients seemed to be nonsignificantly lower with cetuximab treatment than all other treatments for either subgroup. Unfortunately however, late toxicity for *KRAS-*variant patients treated with cetuximab was nonsignificantly worse than for cisplatin-treated *KRAS-*variant patients.

There have been numerous studies on the role of cetuximab in patients with the *KRAS-*variant. Surprisingly, although tumor-acquired *KRAS* mutations predict a poor response to cetuximab, in patients with colon cancer, *KRAS-*variant patients seem to respond to single-agent cetuximab, even in the context of a tumor *KRAS* mutation, without any additional benefit from chemotherapy (10). Importantly, the role of cetuximab in the treatment of *KRAS-*variant patients is strongly affected by how cetuximab is given, such as alone or in combination with other cytotoxic agents. For example, in metastatic colon cancer, cetuximab treatment alone improves survival significantly for these patients (10, 17, 18), but if given in combination with irinotecan, *KRAS-*variant patients had a significantly worse outcome (19).

Two studies have investigated the role of cetuximab in combination with radiation and chemotherapy for *KRAS-*variant patients. The NRG/RTOG 0522 trial included cisplatin with radiation with or without cetuximab in HNSCC (16), and the NRG/RTOG 0617 trial in NSCLC(12) included combination chemotherapy with radiation with or without cetuximab. Both studies suggested that there may be a benefit in outcome for *KRAS-*variant patients with the addition of cetuximab to chemotherapy and radiation. Of note, both of these prior studies also suggested that *KRAS-*variant patients had increased radiosensitivity with chemotherapy and radiation, which in NRG/RTOG 0522 was not worsened with the addition of cetuximab (16) and in NRG/RTOG 0617 was worsened with the addition of cetuximab (12).

This study is the first evaluation of radiation with cetuximab alone in *KRAS-*variant patients. In addition, this study is the only study thus far to evaluate the impact of cetuximab on acute versus late radiation toxicity for *KRAS-*variant patients. This may be an important distinction, as we found here that acute toxicity with cetuximab and radiation versus cisplatin and radiation was numerically, although not significantly, lower for *KRAS-*variant patients. In addition, acute toxicity with cetuximab was lower for *KRAS-*variant patients compared to nonvariant patients treated with either cisplatin or cetuximab. However, there seemed to be higher late toxicity for IMRT + cetuximab–treated *KRAS-*variant patients compared with cisplatin-treated *KRAS-*variant patients. This increased late toxicity for *KRAS-*variant patients could be explained by the significant higher circulating TGFB1 levels previously shown in *KRAS-*variant patients with HNSCC (16), as TGFB1 has been shown to be an essential factor in the development of late toxicity (20).

The different predictive power of the *KRAS-*variant for acute versus late toxicity is both interesting and could be important. In fact, there are now numerous studies showing that the class of germline microRNA-associated mutations (mirSNPs), of which the *KRAS-*variant was the first example, contains powerful biomarkers identifying patients who have different toxicity risks to radiotherapy. The first study showing this was in sarcoma, in which a mirSNP signature identified patients at increased risk of major wound toxicity after steriotactic body radiation therapy (SBRT; ref. 21). Subsequent studies have focused on prostate cancer, in which mirSNP signatures have been found to predict late toxicity to both SBRT and conventionally fractionated radiation therapy CFRT, and the mirSNP signatures differ between the two treatment approaches (22, 23). A recent study also found a mirSNP signature predicting acute toxicity to prostate cancer SBRT that is unique from the signature of late toxicity (Weidhaas, 2025), which further validates the unique genetic responses leading to these two toxicity endpoints. This is consistent with our preliminary findings in this study, showing that *KRAS-*variant patients could experience less acute toxicity but more late toxicity.

Although major limitations of the study include the small number of *KRAS-*variant patients, significantly limiting statistical power, this study is the largest study of HPV+ OPSCC that exists. It is also very likely based on additional mirSNP studies discussed above that radiation toxicity prediction requires more than a single mirSNP, and only the *KRAS-*variant was evaluated in this study. Regardless, although this study does not support the use of the *KRAS-*variant alone in directing cetuximab therapy for patients with HPV+ OPSCC, this study does expand the growing evidence supporting the potential of this class of biomarkers in the personalization of radiotherapy. With patient-specific genetic mirSNP information, there is hope that in the near future we will be able to not only consider patient response to treatment but also their individual acute and late toxicity risks. These are critical steps toward comprehensive treatment personalization for patients being treated with radiotherapy.

## Supplementary Material

Supplementary Table 1 Missing Data Analysis

Supplementary Figure 1 CONSORT flow diagram

Supplementary Table 2 Patient and Tumor Characteristics by KRAS and Assigned Treatment

Supplementary Figure 2 Overall Survival by KRAS

Supplementary Table 3 Univariate and Multivariable Cox Models for KRAS as a Prognostic Biomarker for Overall Survival

Supplementary Figure 3 Progression-Free Survival by KRAS

Supplementary Table 4 Univariate and Multivariable Cox Models for KRAS as a Prognostic Biomarker for Progression-Free Survival

Supplementary Figure 4 Local-Regional Failure by KRAS

Supplementary Table 5 Univariate and Multivariable Cause-Specific Cox Models for KRAS as a Prognostic Biomarker for Local-Regional Failure

Supplementary Figure 5 Distant Metastasis by KRAS

Supplementary Table 6 Univariate and Multivariable Cause-Specific Cox Models for KRAS as a Prognostic Biomarker for Distant Metastasis

Supplementary Figure 6 Overall Survival by KRAS and Assigned Treatment

Supplementary Table 7 Multivariable Cox Models for KRAS as a Predictive Biomarker for Overall Survival

Supplementary Figure 7 Progression-Free Survival by KRAS and Assigned Treatment

Supplementary Table 8 Multivariable Cox Models for KRAS as a Predictive Biomarker for Progression-Free Survival

Supplementary Table 9 Multivariable Cox Models for KRAS as a Predictive Biomarker for Local-Regional Failure

Supplementary Table 10 Multivariable Cox Models for KRAS as a Predictive Biomarker for Distant Metastasis

Supplementary Table 11 Grade 3-4 Treatment-Related [1] Skin Reaction Inside Portal [2] by KRAS and Assigned Treatment

Supplementary Table 12 Grade 3-4 Treatment-Related [1] Skin Reaction Outside Portal [2] by KRAS and Assigned Treatment

Supplementary Table 13 Representativeness of Study Participants

## Data Availability

The data generated in this study are not publicly available as patients did not consent to individual genetic data being shared, but group data are available upon reasonable request to the corresponding author.
