# Supplementary material for: The KRAS-Variant and Cetuximab in HPV-Positive Oropharyngeal Cancer: Results from the NRG/RTOG 1016 Trial
Source: Cancer Res Commun. 2026 Mar 31;6(3):706–13. doi: 10.1158/2767-9764.CRC-25-0551 (PMC13036839; doi:10.1158/2767-9764.CRC-25-0551)
Supplement: Supplementary Table 1 — Missing Data Analysis [file crc-25-0551_supplementary_table_1_suppst1.docx]

**Supplemental Table 1: Missing Data Analysis**

| **Patient and Tumor Characteristics by Inclusion in *KRAS* Analysis** | | |
| --- | --- | --- |
| **Patient or Tumor Characteristic** | **Excluded (n=243)** | **Included (n=562)** |
|  | | |
| Assigned treatment |  |  |
| IMRT + Cisplatin | 131 (53.9%) | 275 (48.9%) |
| IMRT + Cetuximab | 112 (46.1%) | 287 (51.1%) |
|  | | |
| Age (years) |  |  |
| ≤ 49 | 50 (20.6%) | 86 (15.3%) |
| 50 - 59 | 96 (39.5%) | 218 (38.8%) |
| 60 - 69 | 83 (34.2%) | 224 (39.9%) |
| ≥ 70 | 14 (5.8%) | 34 (6.0%) |
| Median | 57 | 59 |
| Min - Max | 36 - 83 | 33 - 82 |
| Q1 - Q3 | 51 - 63 | 52 - 63 |
|  | | |
| Gender |  |  |
| Male | 213 (87.7%) | 515 (91.6%) |
| Female | 30 (12.3%) | 47 (8.4%) |
|  | | |
| Zubrod performance status |  |  |
| 0 | 179 (73.7%) | 416 (74.0%) |
| 1 | 64 (26.3%) | 146 (26.0%) |
|  | | |
| Smoking history |  |  |
| ≤ 10 pack-years | 166 (68.3%) | 336 (59.8%) |
| > 10 pack-years | 77 (31.7%) | 226 (40.2%) |
| Median | 1 | 4 |
| Min - Max | 0 - 92 | 0 - 202 |
| Q1 - Q3 | 0 - 18 | 0 - 26 |
|  | | |
| T stage (AJCC 7th edition) |  |  |
| T1 | 59 (24.3%) | 116 (20.6%) |
| T2 | 91 (37.4%) | 234 (41.6%) |
| T3 | 58 (23.9%) | 150 (26.7%) |
| T4 | 35 (14.4%) | 62 (11.0%) |
|  | | |
| N stage (AJCC 7th edition) |  |  |
| N0 | 9 (3.7%) | 25 (4.4%) |
| N1 | 15 (6.2%) | 30 (5.3%) |
| N2a | 34 (14.0%) | 81 (14.4%) |
| N2b | 117 (48.1%) | 300 (53.4%) |
| N2c | 58 (23.9%) | 107 (19.0%) |
| N3 | 10 (4.1%) | 19 (3.4%) |
|  | | |
| RTOG 0129 risk group* |  |  |
| Low | 179 (73.7%) | 394 (70.1%) |
| Intermediate | 64 (26.3%) | 168 (29.9%) |
|  | | |
| Q1, first quartile; Q3, third quartile; AJCC, American Joint Committee on Cancer. *Low: >10 pack-years and N0-N2a, or ≤10 pack-years; intermediate: >10 pack-years and N2b-N3. | | |
