# Supplementary material for: The KRAS-Variant and Cetuximab in HPV-Positive Oropharyngeal Cancer: Results from the NRG/RTOG 1016 Trial
Source: Cancer Res Commun. 2026 Mar 31;6(3):706–13. doi: 10.1158/2767-9764.CRC-25-0551 (PMC13036839; doi:10.1158/2767-9764.CRC-25-0551)
Supplement: Supplementary Figure 1 — CONSORT flow diagram [file crc-25-0551_supplementary_figure_1_suppsf1.docx]

**Supplemental Figure 1: CONSORT flow diagram**

| Enrolled to RTOG 1016 (n=987) | |
| --- | --- |
| ↓ | |
| Randomized (n=849)  *[not p16-positive (n=50); no tissue submitted for p16 testing (n=7); patient refusal (n=44); physician preference (n=13); disease progression (n=3); other (n=21)]* | |
| ↓ | |
| Included in analysis of primary endpoint (n=805)  *[ineligible (n=44)]* | |
| ↓ | |
| Consented to blood collection (n=733)  *[did not consent (n=72)]* | |
| ↓ | |
| Included in KRAS analysis (n=562)  *[KRAS not assessed (n=171)]* | |
| ↙ ↘ | |
| KRAS-variant (n=92) | Non-variant (n=470) |
