# Supplementary material for: The KRAS-Variant and Cetuximab in HPV-Positive Oropharyngeal Cancer: Results from the NRG/RTOG 1016 Trial
Source: Cancer Res Commun. 2026 Mar 31;6(3):706–13. doi: 10.1158/2767-9764.CRC-25-0551 (PMC13036839; doi:10.1158/2767-9764.CRC-25-0551)
Supplement: Supplementary Table 2 — Patient and Tumor Characteristics by KRAS and Assigned Treatment [file crc-25-0551_supplementary_table_2_suppst2.docx]

| **Supplemental Table 2: Patient and Tumor Characteristics by KRAS and Assigned Treatment** | | | | | | |  |
| --- | --- | --- | --- | --- | --- | --- | --- |
| **Patient or Tumor Characteristic** | **KRAS-variant IMRT + Cisplatin (n=44)** | **KRAS-variant IMRT + Cetuximab (n=48)** | **KRAS-variant Total (n=92)** | **Non-variant IMRT + Cisplatin (n=231)** | **Non-variant IMRT + Cetuximab (n=239)** | **Non-variant Total (n=470)** | |
|  | | | | | | | |
| Age (years) |  |  |  |  |  |  | |
| ≤ 49 | 4 (9.1%) | 8 (16.7%) | 12 (13.0%) | 35 (15.2%) | 39 (16.3%) | 74 (15.7%) | |
| 50 - 59 | 18 (40.9%) | 20 (41.7%) | 38 (41.3%) | 84 (36.4%) | 96 (40.2%) | 180 (38.3%) | |
| 60 - 69 | 20 (45.5%) | 17 (35.4%) | 37 (40.2%) | 94 (40.7%) | 93 (38.9%) | 187 (39.8%) | |
| ≥ 70 | 2 (4.5%) | 3 (6.3%) | 5 (5.4%) | 18 (7.8%) | 11 (4.6%) | 29 (6.2%) | |
| Median | 59.5 | 56 | 57.5 | 59 | 59 | 59 | |
| Min - Max | 45 - 71 | 40 - 72 | 40 - 72 | 33 - 82 | 33 - 80 | 33 - 82 | |
| Q1 - Q3 | 54 - 64 | 51 - 64 | 52 - 64 | 52 - 64 | 53 - 63 | 52 - 63 | |
|  | | | | | | | |
| Gender |  |  |  |  |  |  | |
| Male | 42 ( 95.5%) | 41 ( 85.4%) | 83 ( 90.2%) | 215 ( 93.1%) | 217 ( 90.8%) | 432 ( 91.9%) | |
| Female | 2 ( 4.5%) | 7 ( 14.6%) | 9 ( 9.8%) | 16 ( 6.9%) | 22 ( 9.2%) | 38 ( 8.1%) | |
|  | | | | | | | |
| Zubrod performance status |  |  |  |  |  |  | |
| 0 | 28 ( 63.6%) | 39 ( 81.3%) | 67 ( 72.8%) | 172 ( 74.5%) | 177 ( 74.1%) | 349 ( 74.3%) | |
| 1 | 16 ( 36.4%) | 9 ( 18.8%) | 25 ( 27.2%) | 59 ( 25.5%) | 62 ( 25.9%) | 121 ( 25.7%) | |
|  | | | | | | | |
| Smoking history |  |  |  |  |  |  | |
| ≤ 10 pack-years | 25 ( 56.8%) | 28 ( 58.3%) | 53 ( 57.6%) | 135 ( 58.4%) | 148 ( 61.9%) | 283 ( 60.2%) | |
| > 10 pack-years | 19 ( 43.2%) | 20 ( 41.7%) | 39 ( 42.4%) | 96 ( 41.6%) | 91 ( 38.1%) | 187 ( 39.8%) | |
| Median | 4.5 | 7 | 6 | 5 | 2 | 3.5 | |
| Min - Max | 0 - 108 | 0 - 155 | 0 - 155 | 0 - 147 | 0 - 202 | 0 - 202 | |
| Q1 - Q3 | 0 - 38 | 0 - 29 | 0 - 33 | 0 - 24 | 0 - 24 | 0 - 24 | |
|  | | | | | | | |
| T stage (AJCC 7th edition) |  |  |  |  |  |  | |
| T1 | 8 ( 18.2%) | 11 ( 22.9%) | 19 ( 20.7%) | 42 ( 18.2%) | 55 ( 23.0%) | 97 ( 20.6%) | |
| T2 | 21 ( 47.7%) | 22 ( 45.8%) | 43 ( 46.7%) | 93 ( 40.3%) | 98 ( 41.0%) | 191 ( 40.6%) | |
| T3 | 10 ( 22.7%) | 12 ( 25.0%) | 22 ( 23.9%) | 71 ( 30.7%) | 57 ( 23.8%) | 128 ( 27.2%) | |
| T4 | 5 ( 11.4%) | 3 ( 6.3%) | 8 ( 8.7%) | 25 ( 10.8%) | 29 ( 12.1%) | 54 ( 11.5%) | |
|  | | | | | | | |
| N stage (AJCC 7th edition) |  |  |  |  |  |  | |
| N0 | 0 ( 0.0%) | 1 ( 2.1%) | 1 ( 1.1%) | 13 ( 5.6%) | 11 ( 4.6%) | 24 ( 5.1%) | |
| N1 | 4 ( 9.1%) | 4 ( 8.3%) | 8 ( 8.7%) | 11 ( 4.8%) | 11 ( 4.6%) | 22 ( 4.7%) | |
| N2a | 6 ( 13.6%) | 5 ( 10.4%) | 11 ( 12.0%) | 33 ( 14.3%) | 37 ( 15.5%) | 70 ( 14.9%) | |
| N2b | 24 ( 54.5%) | 26 ( 54.2%) | 50 ( 54.3%) | 120 ( 51.9%) | 130 ( 54.4%) | 250 ( 53.2%) | |
| N2c | 7 ( 15.9%) | 11 ( 22.9%) | 18 ( 19.6%) | 46 ( 19.9%) | 43 ( 18.0%) | 89 ( 18.9%) | |
| N3 | 3 ( 6.8%) | 1 ( 2.1%) | 4 ( 4.3%) | 8 ( 3.5%) | 7 ( 2.9%) | 15 ( 3.2%) | |
|  | | | | | | | |
| RTOG 0129 risk group* |  |  |  |  |  |  | |
| Low | 28 ( 63.6%) | 33 ( 68.8%) | 61 ( 66.3%) | 163 ( 70.6%) | 170 ( 71.1%) | 333 ( 70.9%) | |
| Intermediate | 16 ( 36.4%) | 15 ( 31.3%) | 31 ( 33.7%) | 68 ( 29.4%) | 69 ( 28.9%) | 137 ( 29.1%) | |
|  | | | | | | | |
| Q1, first quartile; Q3, third quartile; AJCC, American Joint Committee on Cancer. *Low: >10 pack-years and N0-N2a, or ≤10 pack-years; intermediate: >10 pack-years and N2b-N3. | | | | | | | |
