# Supplementary figures and images for: The KRAS-Variant and Cetuximab in HPV-Positive Oropharyngeal Cancer: Results from the NRG/RTOG 1016 Trial
Source: Cancer Res Commun. 2026 Mar 31;6(3):706–13. doi: 10.1158/2767-9764.CRC-25-0551 (PMC13036839; doi:10.1158/2767-9764.CRC-25-0551)

**Supplemental Figure 2: Overall Survival by *KRAS***


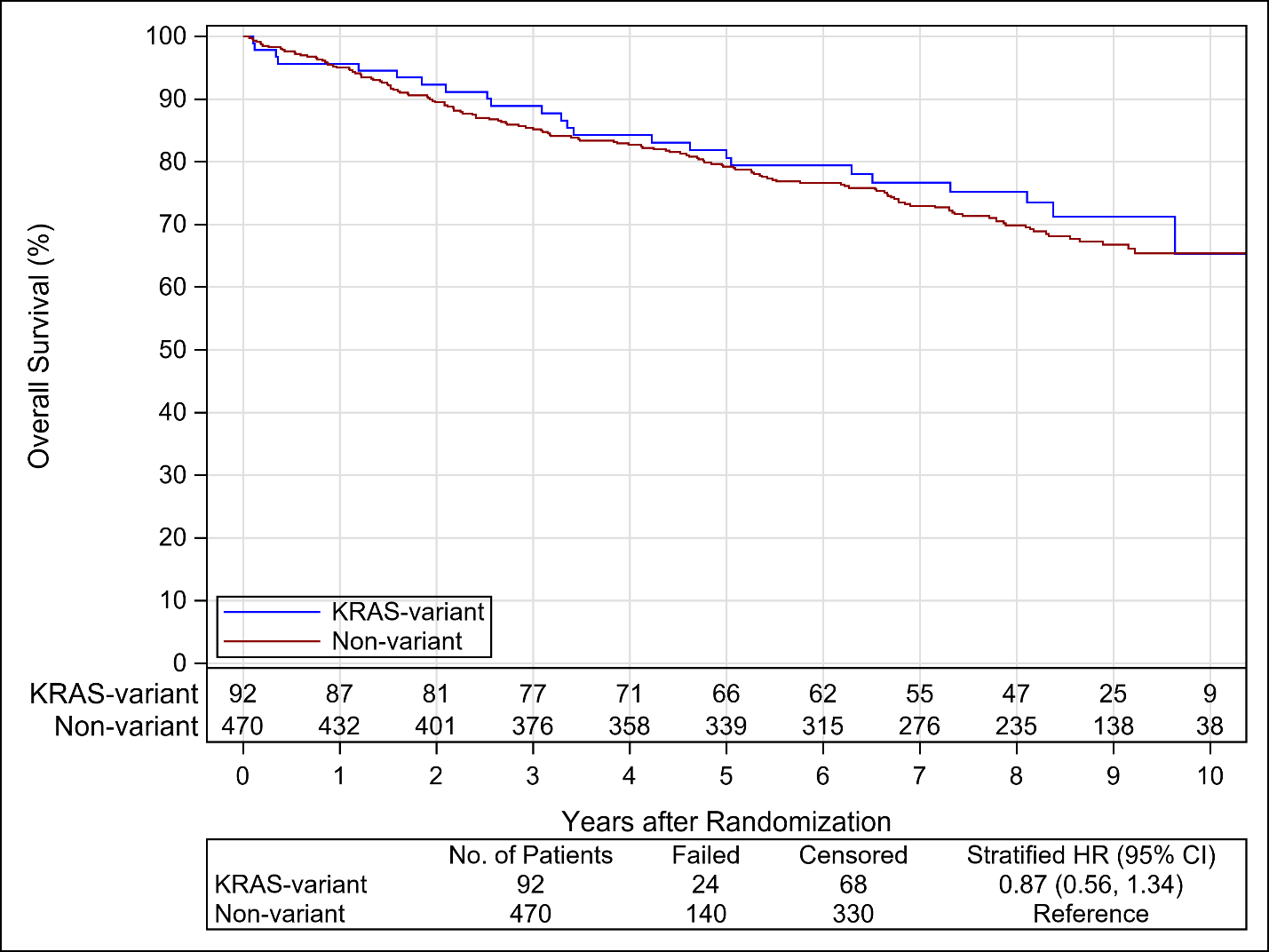

Supplement: Supplementary Figure 2 — Overall Survival by KRAS [file crc-25-0551_supplementary_figure_2_suppsf2.docx]

**Supplemental Figure 3: Progression-Free Survival by *KRAS***


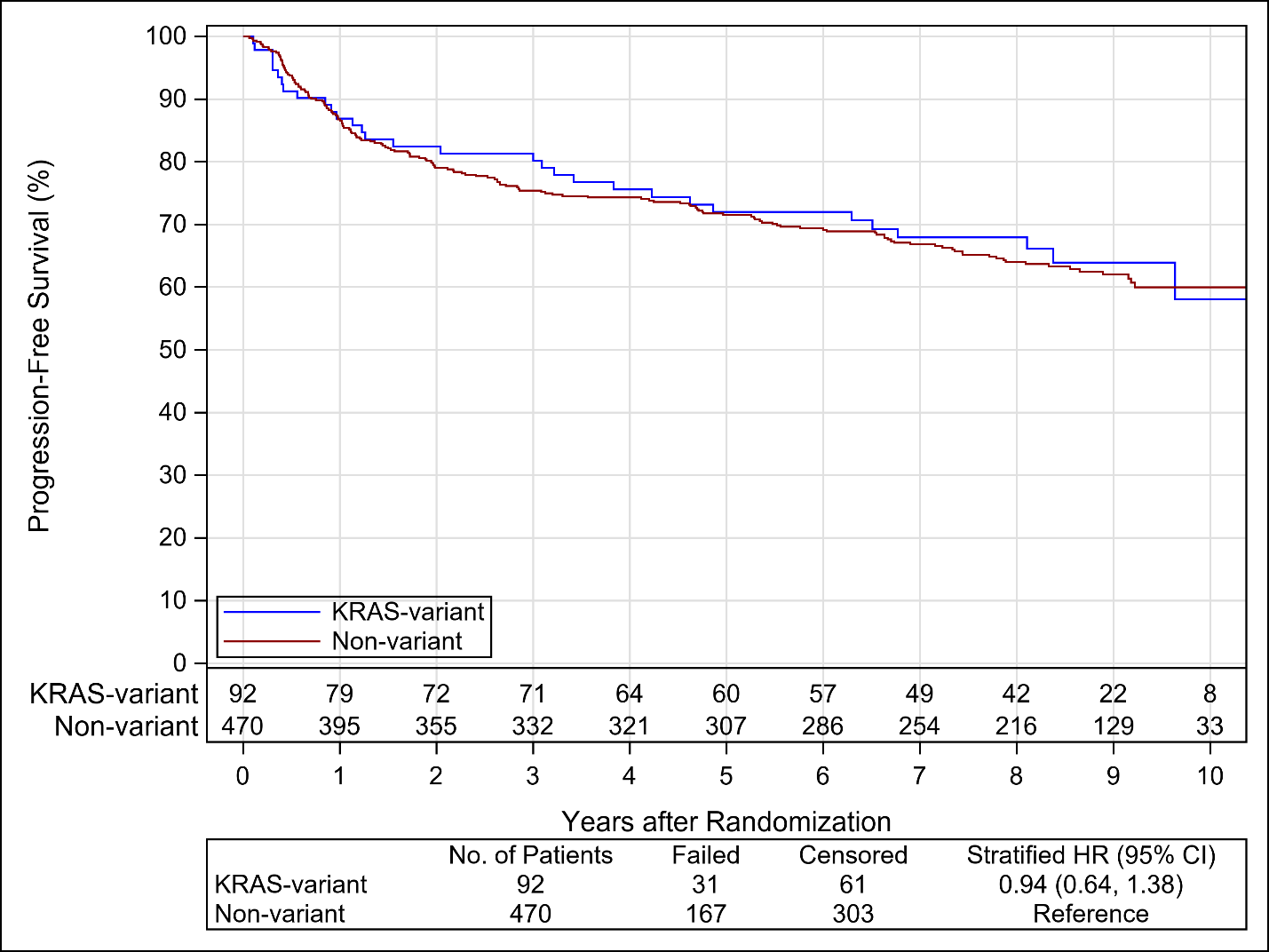

Supplement: Supplementary Figure 3 — Progression-Free Survival by KRAS [file crc-25-0551_supplementary_figure_3_suppsf3.docx]

**Supplemental Figure 4: Local-Regional Failure by *KRAS***


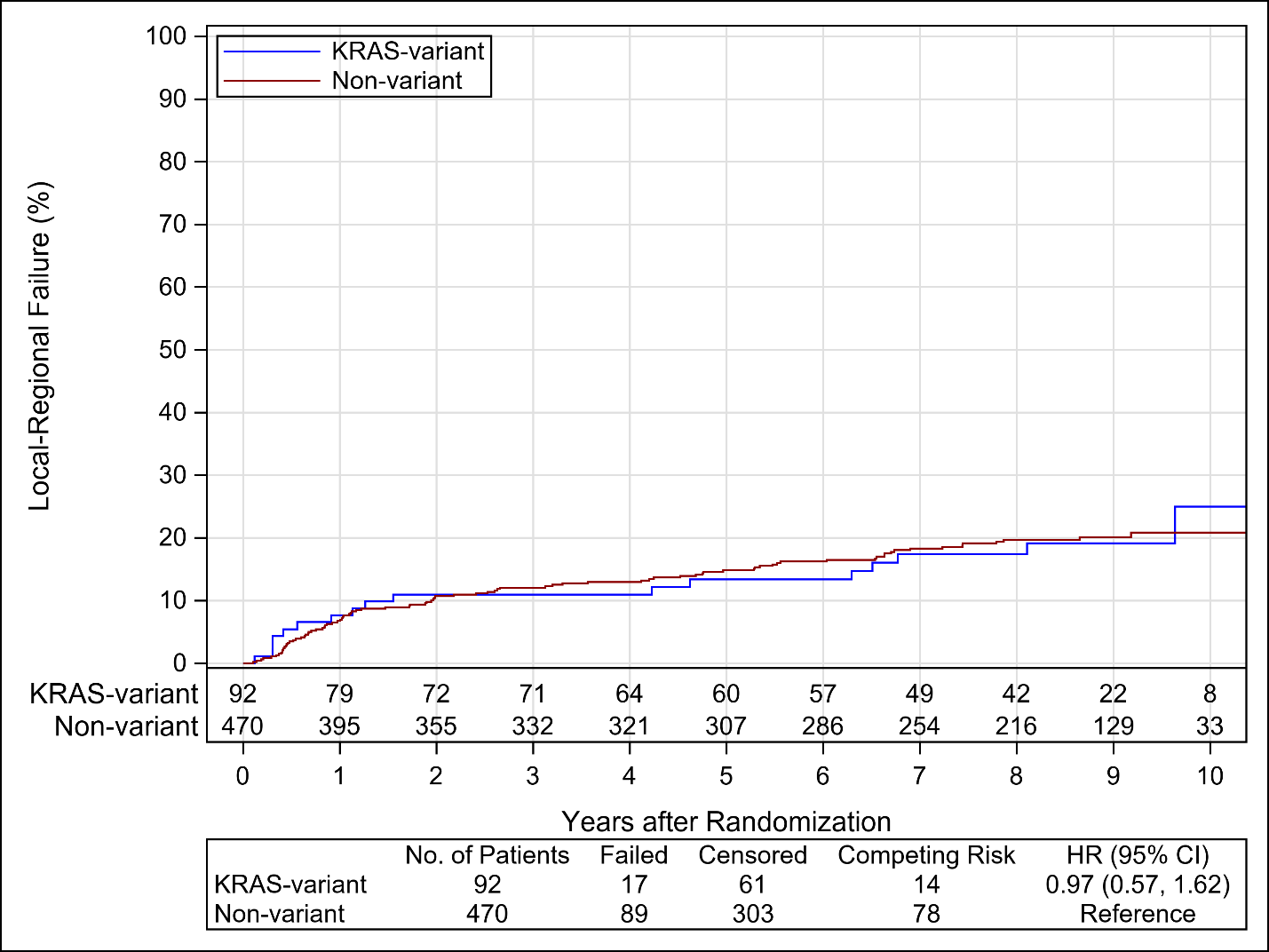

Supplement: Supplementary Figure 4 — Local-Regional Failure by KRAS [file crc-25-0551_supplementary_figure_4_suppsf4.docx]

**Supplemental Figure 5: Distant Metastasis by *KRAS***


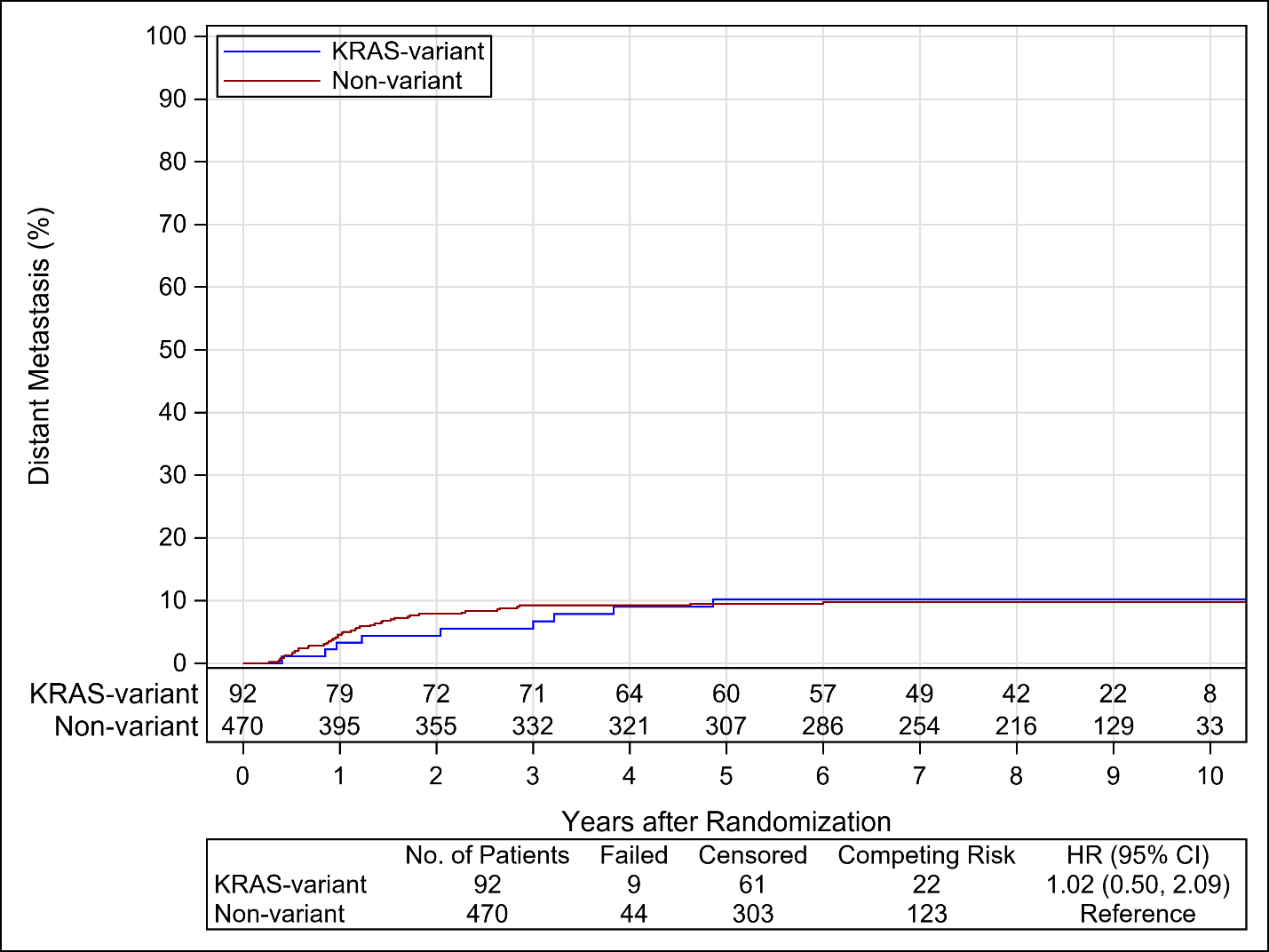

Supplement: Supplementary Figure 5 — Distant Metastasis by KRAS [file crc-25-0551_supplementary_figure_5_suppsf5.docx]

**Supplemental Figure 6: Overall Survival by *KRAS* and Assigned Treatment**


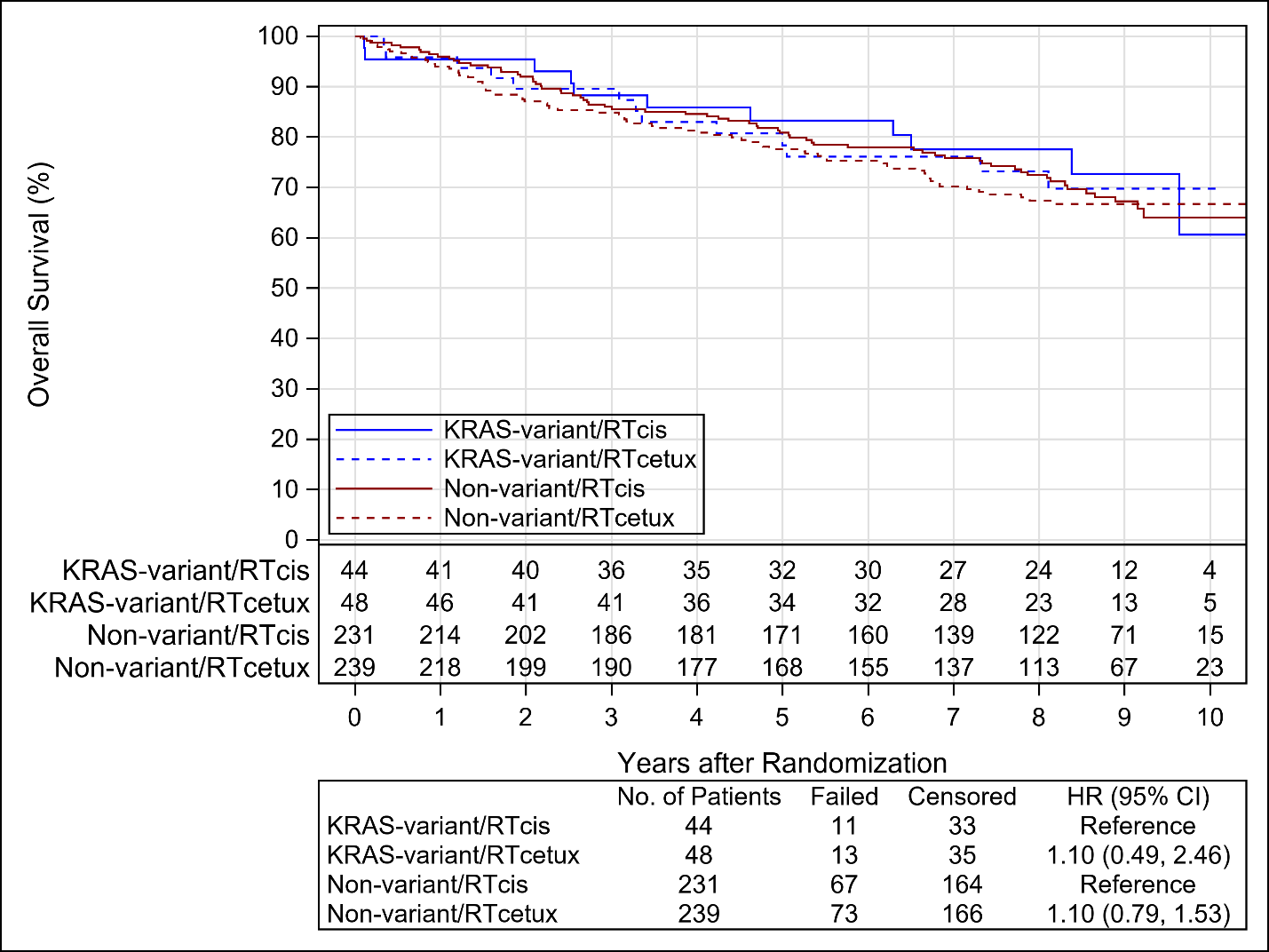

Supplement: Supplementary Figure 6 — Overall Survival by KRAS and Assigned Treatment [file crc-25-0551_supplementary_figure_6_suppsf6.docx]

**Supplemental Figure 7: Progression-Free Survival by *KRAS* and Assigned Treatment**


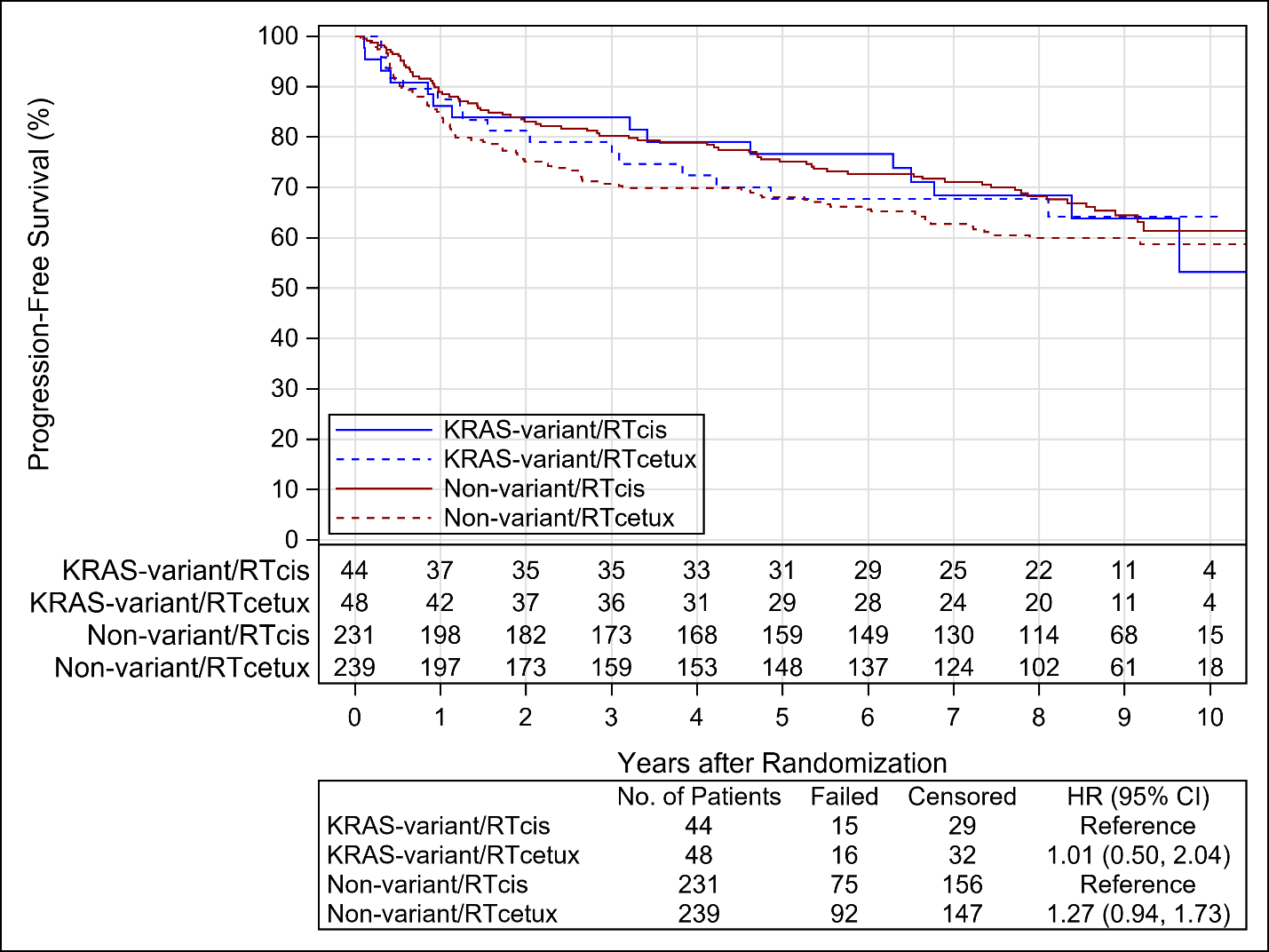

Supplement: Supplementary Figure 7 — Progression-Free Survival by KRAS and Assigned Treatment [file crc-25-0551_supplementary_figure_7_suppsf7.docx]
