# Supplementary material for: The KRAS-Variant and Cetuximab in HPV-Positive Oropharyngeal Cancer: Results from the NRG/RTOG 1016 Trial
Source: Cancer Res Commun. 2026 Mar 31;6(3):706–13. doi: 10.1158/2767-9764.CRC-25-0551 (PMC13036839; doi:10.1158/2767-9764.CRC-25-0551)
Supplement: Supplementary Table 3 — Univariate and Multivariable Cox Models for KRAS as a Prognostic Biomarker for Overall Survival [file crc-25-0551_supplementary_table_3_suppst3.docx]

| **Supplemental Table 3: Univariate and Multivariable Cox Models for KRAS as a Prognostic Biomarker for Overall Survival (n=562; 164 events)** | | | |
| --- | --- | --- | --- |
|  | | **Multivariable** | |
| **Variable** | **Univariate models p-value HR (95% CI)** | **Full model p-value HR (95% CI)** | **Reduced model p-value HR (95% CI)** |
|  | | | |
| KRAS | 0.5142 (1-S 0.2571) | 0.4274 (1-S 0.2137) | 0.3176 (1-S 0.1588) |
| Non-variant | Reference | Reference | Reference |
| KRAS-variant | 0.87 (0.56, 1.34) | 0.84 (0.54, 1.30) | 0.80 (0.52, 1.24) |
|  | | | |
| Age (years) | 0.1116 | 0.5610 |  |
| Continuous, per 1-year increment | 1.016 (0.996, 1.037) | 1.006 (0.986, 1.027) |  |
|  | | | |
| Gender | 0.1798 | 0.1065 |  |
| Female | Reference | Reference |  |
| Male | 1.55 (0.82, 2.94) | 1.71 (0.89, 3.28) |  |
|  | | | |
| Zubrod performance status | 0.0002 | 0.0320 | 0.0193 |
| 0 | Reference | Reference | Reference |
| 1 | 1.84 (1.33, 2.53) | 1.45 (1.03, 2.03) | 1.49 (1.07, 2.07) |
|  | | | |
| Smoking history | 0.0162 | 0.6861 |  |
| ≤ 10 pack-years | Reference | Reference |  |
| > 10 pack-years | 1.46 (1.07, 1.99) | 0.88 (0.47, 1.64) |  |
|  | | | |
| T stage (AJCC 7th edition) | <.0001 | 0.0001 | 0.0002 |
| T1 | Reference | Reference | Reference |
| T2-T3 | 1.95 (1.20, 3.17) | 1.75 (1.07, 2.85) | 1.79 (1.10, 2.92) |
| T4 | 4.42 (2.51, 7.78) | 3.39 (1.88, 6.10) | 3.35 (1.87, 5.99) |
|  | | | |
| N stage (AJCC 7th edition) | <.0001 | <.0001 | <.0001 |
| N0-N2b | Reference | Reference | Reference |
| N2c-N3 | 2.26 (1.64, 3.12) | 1.94 (1.39, 2.71) | 1.93 (1.39, 2.67) |
|  | | | |
| RTOG 0129 risk group* | 0.0013 | 0.0989 | 0.0118 |
| Low | Reference | Reference | Reference |
| Intermediate | 1.67 (1.22, 2.29) | 1.71 (0.90, 3.22) | 1.50 (1.09, 2.07) |
|  | | | |
| Bayesian Information Criterion (BIC) |  | 1721.781 | 1710.200 |
|  | | | |
| HR, hazard ratio; CI, confidence interval; 1-S, one-sided; AJCC, American Joint Committee on Cancer. All p-values are two-sided except where noted. All models are stratified by assigned treatment. *Low: >10 pack-years and N0-N2a, or ≤10 pack-years; intermediate: >10 pack-years and N2b-N3. | | | |
