# Supplementary material for: The KRAS-Variant and Cetuximab in HPV-Positive Oropharyngeal Cancer: Results from the NRG/RTOG 1016 Trial
Source: Cancer Res Commun. 2026 Mar 31;6(3):706–13. doi: 10.1158/2767-9764.CRC-25-0551 (PMC13036839; doi:10.1158/2767-9764.CRC-25-0551)
Supplement: Supplementary Table 4 — Univariate and Multivariable Cox Models for KRAS as a Prognostic Biomarker for Progression-Free Survival [file crc-25-0551_supplementary_table_4_suppst4.docx]

| **Supplemental Table 4: Univariate and Multivariable Cox Models for KRAS as a Prognostic Biomarker for Progression-Free Survival (n=562; 198 events)** | | | |
| --- | --- | --- | --- |
|  | | **Multivariable** | |
| **Variable** | **Univariate models p-value HR (95% CI)** | **Full model p-value HR (95% CI)** | **Reduced model p-value HR (95% CI)** |
|  | | | |
| KRAS | 0.7408 (1-S 0.3704) | 0.6466 (1-S 0.3233) | 0.5549 (1-S 0.2775) |
| Non-variant | Reference | Reference | Reference |
| KRAS-variant | 0.94 (0.64, 1.38) | 0.91 (0.62, 1.34) | 0.89 (0.61, 1.31) |
|  | | | |
| Age (years) | 0.0558 | 0.4315 |  |
| Continuous, per 1-year increment | 1.018 (1.000, 1.036) | 1.008 (0.989, 1.027) |  |
|  | | | |
| Gender | 0.0556 | 0.0377 |  |
| Female | Reference | Reference |  |
| Male | 1.81 (0.99, 3.33) | 1.92 (1.04, 3.56) |  |
|  | | | |
| Zubrod performance status | <.0001 | 0.0081 | 0.0026 |
| 0 | Reference | Reference | Reference |
| 1 | 1.88 (1.40, 2.52) | 1.51 (1.11, 2.06) | 1.59 (1.18, 2.15) |
|  | | | |
| Smoking history | 0.0352 | 0.6950 |  |
| ≤ 10 pack-years | Reference | Reference |  |
| > 10 pack-years | 1.35 (1.02, 1.79) | 0.90 (0.53, 1.52) |  |
|  | | | |
| T stage (AJCC 7th edition) | <.0001 | <.0001 | <.0001 |
| T1 | Reference | Reference | Reference |
| T2-T3 | 2.10 (1.35, 3.28) | 1.91 (1.22, 2.99) | 1.99 (1.27, 3.10) |
| T4 | 4.21 (2.49, 7.12) | 3.36 (1.94, 5.80) | 3.35 (1.95, 5.74) |
|  | | | |
| N stage (AJCC 7th edition) | 0.0002 | 0.0063 | 0.0036 |
| N0-N2b | Reference | Reference | Reference |
| N2c-N3 | 1.78 (1.31, 2.41) | 1.55 (1.13, 2.12) | 1.58 (1.16, 2.15) |
|  | | | |
| RTOG 0129 risk group* | 0.0088 | 0.1478 |  |
| Low | Reference | Reference |  |
| Intermediate | 1.47 (1.10, 1.97) | 1.49 (0.87, 2.57) |  |
|  | | | |
| Bayesian Information Criterion (BIC) |  | 2087.370 | 2076.240 |
|  | | | |
| HR, hazard ratio; CI, confidence interval; 1-S, one-sided; AJCC, American Joint Committee on Cancer. All p-values are two-sided except where noted. All models are stratified by assigned treatment. *Low: >10 pack-years and N0-N2a, or ≤10 pack-years; intermediate: >10 pack-years and N2b-N3. | | | |
