# Supplementary material for: The KRAS-Variant and Cetuximab in HPV-Positive Oropharyngeal Cancer: Results from the NRG/RTOG 1016 Trial
Source: Cancer Res Commun. 2026 Mar 31;6(3):706–13. doi: 10.1158/2767-9764.CRC-25-0551 (PMC13036839; doi:10.1158/2767-9764.CRC-25-0551)
Supplement: Supplementary Table 5 — Univariate and Multivariable Cause-Specific Cox Models for KRAS as a Prognostic Biomarker for Local-Regional Failure [file crc-25-0551_supplementary_table_5_suppst5.docx]

| **Supplemental Table 5: Univariate and Multivariable Cause-Specific Cox Models for KRAS as a Prognostic Biomarker for Local-Regional Failure (n=562; 106 events)** | | | |
| --- | --- | --- | --- |
|  | | **Multivariable** | |
| **Variable** | **Univariate models p-value HR (95% CI)** | **Full model p-value HR (95% CI)** | **Reduced model p-value HR (95% CI)** |
|  | | | |
| KRAS | 0.8941 (1-S 0.4471) | 0.9557 (1-S 0.5222) | 0.8782 (1-S 0.5609) |
| Non-variant | Reference | Reference | Reference |
| KRAS-variant | 0.97 (0.57, 1.62) | 1.02 (0.60, 1.72) | 1.04 (0.62, 1.76) |
|  | | | |
| Age (years) | 0.6852 | 0.5012 |  |
| Continuous, per 1-year increment | 1.005 (0.981, 1.030) | 0.991 (0.966, 1.017) |  |
|  | | | |
| Gender | 0.2825 | 0.1444 |  |
| Female | Reference | Reference |  |
| Male | 1.52 (0.71, 3.28) | 1.79 (0.82, 3.93) |  |
|  | | | |
| Zubrod performance status | <.0001 | 0.0084 | 0.0041 |
| 0 | Reference | Reference | Reference |
| 1 | 2.26 (1.53, 3.35) | 1.76 (1.16, 2.67) | 1.82 (1.21, 2.73) |
|  | | | |
| Smoking history (pack-years) | 0.0955 | 0.6674 |  |
| Continuous, per 1-year increment | 1.005 (0.999, 1.011) | 1.002 (0.993, 1.011) |  |
|  | | | |
| T stage (AJCC 7th edition) | <.0001 | <.0001 | <.0001 |
| T1-T2 | Reference | Reference | Reference |
| T3 | 1.77 (1.13, 2.78) | 1.59 (1.01, 2.53) | 1.61 (1.02, 2.55) |
| T4 | 4.00 (2.46, 6.50) | 3.40 (2.03, 5.70) | 3.37 (2.04, 5.58) |
|  | | | |
| N stage (AJCC 7th edition) | 0.0082 | 0.1339 |  |
| N0-N2b | Reference | Reference |  |
| N2c-N3 | 1.75 (1.16, 2.66) | 1.39 (0.90, 2.14) |  |
|  | | | |
| RTOG 0129 risk group* | 0.0796 | 0.3009 |  |
| Low | Reference | Reference |  |
| Intermediate | 1.43 (0.96, 2.13) | 1.30 (0.79, 2.15) |  |
|  | | | |
| Bayesian Information Criterion (BIC) |  | 1116.863 | 1101.090 |
|  | | | |
| HR, hazard ratio; CI, confidence interval; 1-S, one-sided; AJCC, American Joint Committee on Cancer. All p-values are two-sided except where noted. All models are stratified by assigned treatment. *Low: >10 pack-years and N0-N2a, or ≤10 pack-years; intermediate: >10 pack-years and N2b-N3. | | | |
