# Supplementary material for: The KRAS-Variant and Cetuximab in HPV-Positive Oropharyngeal Cancer: Results from the NRG/RTOG 1016 Trial
Source: Cancer Res Commun. 2026 Mar 31;6(3):706–13. doi: 10.1158/2767-9764.CRC-25-0551 (PMC13036839; doi:10.1158/2767-9764.CRC-25-0551)
Supplement: Supplementary Table 6 — Univariate and Multivariable Cause-Specific Cox Models for KRAS as a Prognostic Biomarker for Distant Metastasis [file crc-25-0551_supplementary_table_6_suppst6.docx]

| **Supplemental Table 6: Univariate and Multivariable Cause-Specific Cox Models for KRAS as a Prognostic Biomarker for Distant Metastasis (n=562; 53 events)** | | | |
| --- | --- | --- | --- |
|  | | **Multivariable** | |
| **Variable** | **Univariate models p-value HR (95% CI)** | **Full model p-value HR (95% CI)** | **Reduced model p-value HR (95% CI)** |
|  | | | |
| KRAS | 0.9543 (1-S 0.5229) | 0.9733 (1-S 0.4867) | 0.9203 (1-S 0.4602) |
| Non-variant | Reference | Reference | Reference |
| KRAS-variant | 1.02 (0.50, 2.09) | 0.99 (0.48, 2.03) | 0.96 (0.47, 1.98) |
|  | | | |
| Age (years) | 0.3433 | 0.5425 |  |
| Continuous, per 1-year increment | 1.017 (0.982, 1.053) | 1.011 (0.976, 1.048) |  |
|  | | | |
| Gender | 0.1796 | 0.1983 |  |
| Female | Reference | Reference |  |
| Male | 2.63 (0.64, 10.82) | 2.54 (0.61, 10.51) |  |
|  | | | |
| Zubrod performance status | 0.1297 | 0.3325 |  |
| 0 | Reference | Reference |  |
| 1 | 1.56 (0.88, 2.79) | 1.34 (0.74, 2.43) |  |
|  | | | |
| Smoking history (pack-years) | 0.4770 | 0.8802 |  |
| Continuous, per 1-year increment | 1.003 (0.994, 1.013) | 1.001 (0.989, 1.013) |  |
|  | | | |
| T stage (AJCC 7th edition) | 0.0722 | 0.1433 |  |
| T1 | Reference | Reference |  |
| T2-T4 | 2.08 (0.94, 4.61) | 1.83 (0.82, 4.09) |  |
|  | | | |
| N stage (AJCC 7th edition) | 0.0066 | 0.0116 | 0.0066 |
| N0-N2b | Reference | Reference | Reference |
| N2c-N3 | 2.18 (1.24, 3.82) | 2.09 (1.18, 3.69) | 2.18 (1.24, 3.83) |
|  | | | |
| RTOG 0129 risk group* | 0.6090 | 0.9483 |  |
| Low | Reference | Reference |  |
| Intermediate | 1.16 (0.65, 2.07) | 1.02 (0.49, 2.14) |  |
|  | | | |
| Bayesian Information Criterion (BIC) |  | 594.120 | 577.613 |
|  | | | |
| HR, hazard ratio; CI, confidence interval; 1-S, one-sided; AJCC, American Joint Committee on Cancer. All p-values are two-sided except where noted. All models are stratified by assigned treatment. *Low: >10 pack-years and N0-N2a, or ≤10 pack-years; intermediate: >10 pack-years and N2b-N3. | | | |
