# Supplementary material for: The KRAS-Variant and Cetuximab in HPV-Positive Oropharyngeal Cancer: Results from the NRG/RTOG 1016 Trial
Source: Cancer Res Commun. 2026 Mar 31;6(3):706–13. doi: 10.1158/2767-9764.CRC-25-0551 (PMC13036839; doi:10.1158/2767-9764.CRC-25-0551)
Supplement: Supplementary Table 7 — Multivariable Cox Models for KRAS as a Predictive Biomarker for Overall Survival [file crc-25-0551_supplementary_table_7_suppst7.docx]

| **Supplemental Table 7: Multivariable Cox Models for KRAS as a Predictive Biomarker for Overall Survival (n=562; 164 events)** | | | |
| --- | --- | --- | --- |
| **Variable** | **Base model p-value HR (95% CI)** | **Full model p-value HR (95% CI)** | **Reduced model p-value HR (95% CI)** |
|  | | | |
| KRAS X assigned treatment interaction | 0.9890 | 0.5718 | 0.6540 |
|  | | | |
| KRAS |  |  |  |
| If IMRT + Cisplatin: |  |  |  |
| Non-variant | Reference | Reference | Reference |
| KRAS-variant | 0.86 (0.46, 1.64) | 0.74 (0.39, 1.41) | 0.73 (0.38, 1.39) |
| If IMRT + Cetuximab: |  |  |  |
| Non-variant | Reference | Reference | Reference |
| KRAS-variant | 0.87 (0.48, 1.57) | 0.96 (0.52, 1.74) | 0.89 (0.49, 1.61) |
|  | | | |
| Assigned treatment |  |  |  |
| If Non-variant: |  |  |  |
| IMRT + Cisplatin | Reference | Reference | Reference |
| IMRT + Cetuximab | 1.10 (0.79, 1.53) | 1.18 (0.84, 1.64) | 1.20 (0.86, 1.67) |
| If KRAS-variant: |  |  |  |
| IMRT + Cisplatin | Reference | Reference | Reference |
| IMRT + Cetuximab | 1.10 (0.49, 2.46) | 1.52 (0.67, 3.44) | 1.46 (0.65, 3.32) |
|  | | | |
| Age (years) |  | 0.5600 |  |
| Continuous, per 1-year increment |  | 1.006 (0.986, 1.027) |  |
|  | | | |
| Gender |  | 0.1080 |  |
| Female |  | Reference |  |
| Male |  | 1.70 (0.89, 3.26) |  |
|  | | | |
| Zubrod performance status |  | 0.0258 | 0.0157 |
| 0 |  | Reference | Reference |
| 1 |  | 1.47 (1.05, 2.06) | 1.51 (1.08, 2.10) |
|  | | | |
| Smoking history |  | 0.6388 |  |
| ≤ 10 pack-years |  | Reference |  |
| > 10 pack-years |  | 0.86 (0.46, 1.61) |  |
|  | | | |
| T stage (AJCC 7th edition) |  | <.0001 | <.0001 |
| T1 |  | Reference | Reference |
| T2-T3 |  | 1.75 (1.07, 2.87) | 1.79 (1.10, 2.93) |
| T4 |  | 3.49 (1.94, 6.29) | 3.45 (1.93, 6.16) |
|  | | | |
| N stage (AJCC 7th edition) |  | <.0001 | <.0001 |
| N0-N2b |  | Reference | Reference |
| N2c-N3 |  | 1.94 (1.39, 2.71) | 1.94 (1.40, 2.69) |
|  | | | |
| RTOG 0129 risk group* |  | 0.0813 | 0.0092 |
| Low |  | Reference | Reference |
| Intermediate |  | 1.76 (0.93, 3.33) | 1.53 (1.11, 2.10) |
|  | | | |
| Bayesian Information Criterion (BIC) | 1976.352 | 1953.888 | 1942.346 |
|  | | | |
| HR, hazard ratio; CI, confidence interval; AJCC, American Joint Committee on Cancer. *Low: >10 pack-years and N0-N2a, or ≤10 pack-years; intermediate: >10 pack-years and N2b-N3. | | | |
