# Supplementary material for: The KRAS-Variant and Cetuximab in HPV-Positive Oropharyngeal Cancer: Results from the NRG/RTOG 1016 Trial
Source: Cancer Res Commun. 2026 Mar 31;6(3):706–13. doi: 10.1158/2767-9764.CRC-25-0551 (PMC13036839; doi:10.1158/2767-9764.CRC-25-0551)
Supplement: Supplementary Table 8 — Multivariable Cox Models for KRAS as a Predictive Biomarker for Progression-Free Survival [file crc-25-0551_supplementary_table_8_suppst8.docx]

| **Supplemental Table 8: Multivariable Cox Models for KRAS as a Predictive Biomarker for Progression-Free Survival (n=562; 198 events)** | | | |
| --- | --- | --- | --- |
| **Variable** | **Base model p-value HR (95% CI)** | **Full model p-value HR (95% CI)** | **Reduced model p-value HR (95% CI)** |
|  | | | |
| KRAS X assigned treatment interaction | 0.5556 | 0.9514 | 0.7700 |
|  | | | |
| KRAS |  |  |  |
| If IMRT + Cisplatin: |  |  |  |
| Non-variant | Reference | Reference | Reference |
| KRAS-variant | 1.06 (0.61, 1.85) | 0.93 (0.53, 1.63) | 0.95 (0.54, 1.66) |
| If IMRT + Cetuximab: |  |  |  |
| Non-variant | Reference | Reference | Reference |
| KRAS-variant | 0.85 (0.50, 1.44) | 0.91 (0.53, 1.55) | 0.85 (0.50, 1.45) |
|  | | | |
| Assigned treatment |  |  |  |
| If Non-variant: |  |  |  |
| IMRT + Cisplatin | Reference | Reference | Reference |
| IMRT + Cetuximab | 1.27 (0.94, 1.73) | 1.39 (1.02, 1.89) | 1.40 (1.03, 1.90) |
| If KRAS-variant: |  |  |  |
| IMRT + Cisplatin | Reference | Reference | Reference |
| IMRT + Cetuximab | 1.01 (0.50, 2.04) | 1.36 (0.66, 2.78) | 1.24 (0.61, 2.54) |
|  | | | |
| Age (years) |  | 0.4690 |  |
| Continuous, per 1-year increment |  | 1.007 (0.988, 1.026) |  |
|  | | | |
| Gender |  | 0.0403 |  |
| Female |  | Reference |  |
| Male |  | 1.91 (1.03, 3.53) |  |
|  | | | |
| Zubrod performance status |  | 0.0068 | 0.0024 |
| 0 |  | Reference | Reference |
| 1 |  | 1.53 (1.12, 2.08) | 1.60 (1.18, 2.16) |
|  | | | |
| Smoking history |  | 0.6681 |  |
| ≤ 10 pack-years |  | Reference |  |
| > 10 pack-years |  | 0.89 (0.53, 1.51) |  |
|  | | | |
| T stage (AJCC 7th edition) |  | <.0001 | <.0001 |
| T1 |  | Reference | Reference |
| T2-T3 |  | 1.94 (1.24, 3.03) | 2.01 (1.29, 3.14) |
| T4 |  | 3.43 (1.99, 5.93) | 3.41 (1.99, 5.84) |
|  | | | |
| N stage (AJCC 7th edition) |  | 0.0058 | 0.0029 |
| N0-N2b |  | Reference | Reference |
| N2c-N3 |  | 1.56 (1.14, 2.13) | 1.59 (1.17, 2.17) |
|  | | | |
| RTOG 0129 risk group* |  | 0.1368 |  |
| Low |  | Reference |  |
| Intermediate |  | 1.51 (0.88, 2.61) |  |
|  | | | |
| Bayesian Information Criterion (BIC) | 2380.741 | 2363.977 | 2352.717 |
|  | | | |
| HR, hazard ratio; CI, confidence interval; AJCC, American Joint Committee on Cancer. *Low: >10 pack-years and N0-N2a, or ≤10 pack-years; intermediate: >10 pack-years and N2b-N3. | | | |
