# Supplementary material for: The KRAS-Variant and Cetuximab in HPV-Positive Oropharyngeal Cancer: Results from the NRG/RTOG 1016 Trial
Source: Cancer Res Commun. 2026 Mar 31;6(3):706–13. doi: 10.1158/2767-9764.CRC-25-0551 (PMC13036839; doi:10.1158/2767-9764.CRC-25-0551)
Supplement: Supplementary Table 9 — Multivariable Cox Models for KRAS as a Predictive Biomarker for Local-Regional Failure [file crc-25-0551_supplementary_table_9_suppst9.docx]

| **Supplemental Table 9: Multivariable Cox Models for KRAS as a Predictive Biomarker for Local-Regional Failure (n=562; 106 events)** | | | |
| --- | --- | --- | --- |
| **Variable** | **Base model p-value HR (95% CI)** | **Full model p-value HR (95% CI)** | **Reduced model p-value HR (95% CI)** |
|  | | | |
| KRAS X assigned treatment interaction | 0.0884 | 0.2006 | 0.1933 |
|  | | | |
| KRAS |  |  |  |
| If IMRT + Cisplatin: |  |  |  |
| Non-variant | Reference | Reference | Reference |
| KRAS-variant | 1.56 (0.77, 3.17) | 1.46 (0.71, 2.98) | 1.50 (0.74, 3.05) |
| If IMRT + Cetuximab: |  |  |  |
| Non-variant | Reference | Reference | Reference |
| KRAS-variant | 0.62 (0.28, 1.37) | 0.72 (0.33, 1.61) | 0.74 (0.34, 1.63) |
|  | | | |
| Assigned treatment |  |  |  |
| If Non-variant: |  |  |  |
| IMRT + Cisplatin | Reference | Reference | Reference |
| IMRT + Cetuximab | 1.67 (1.09, 2.57) | 1.89 (1.23, 2.91) | 1.83 (1.19, 2.82) |
| If KRAS-variant: |  |  |  |
| IMRT + Cisplatin | Reference | Reference | Reference |
| IMRT + Cetuximab | 0.67 (0.25, 1.75) | 0.94 (0.35, 2.51) | 0.90 (0.34, 2.41) |
|  | | | |
| Age (years) |  | 0.4589 |  |
| Continuous, per 1-year increment |  | 0.990 (0.965, 1.016) |  |
|  | | | |
| Gender |  | 0.1464 |  |
| Female |  | Reference |  |
| Male |  | 1.79 (0.82, 3.93) |  |
|  | | | |
| Zubrod performance status |  | 0.0103 | 0.0055 |
| 0 |  | Reference | Reference |
| 1 |  | 1.73 (1.14, 2.64) | 1.79 (1.19, 2.71) |
|  | | | |
| Smoking history (pack-years) |  | 0.6330 |  |
| Continuous, per 1-year increment |  | 1.002 (0.993, 1.011) |  |
|  | | | |
| T stage (AJCC 7th edition) |  | <.0001 | <.0001 |
| T1-T2 |  | Reference | Reference |
| T3 |  | 1.58 (1.00, 2.50) | 1.60 (1.02, 2.52) |
| T4 |  | 3.38 (2.01, 5.66) | 3.34 (2.02, 5.53) |
|  | | | |
| N stage (AJCC 7th edition) |  | 0.1174 |  |
| N0-N2b |  | Reference |  |
| N2c-N3 |  | 1.41 (0.92, 2.17) |  |
|  | | | |
| RTOG 0129 risk group* |  | 0.3198 |  |
| Low |  | Reference |  |
| Intermediate |  | 1.29 (0.78, 2.13) |  |
|  | | | |
| Bayesian Information Criterion (BIC) | 1270.917 | 1266.019 | 1250.391 |
|  | | | |
| HR, hazard ratio; CI, confidence interval; AJCC, American Joint Committee on Cancer. *Low: >10 pack-years and N0-N2a, or ≤10 pack-years; intermediate: >10 pack-years and N2b-N3. | | | |
