# Supplementary material for: The KRAS-Variant and Cetuximab in HPV-Positive Oropharyngeal Cancer: Results from the NRG/RTOG 1016 Trial
Source: Cancer Res Commun. 2026 Mar 31;6(3):706–13. doi: 10.1158/2767-9764.CRC-25-0551 (PMC13036839; doi:10.1158/2767-9764.CRC-25-0551)
Supplement: Supplementary Table 10 — Multivariable Cox Models for KRAS as a Predictive Biomarker for Distant Metastasis [file crc-25-0551_supplementary_table_10_suppst10.docx]

| **Supplemental Table 10: Multivariable Cox Models for KRAS as a Predictive Biomarker for Distant Metastasis (n=562; 53 events)** | | | |
| --- | --- | --- | --- |
| **Variable** | **Base model p-value HR (95% CI)** | **Full model p-value HR (95% CI)** | **Reduced model p-value HR (95% CI)** |
|  | | | |
| KRAS X assigned treatment interaction | 0.1878 | 0.1560 | 0.2160 |
|  | | | |
| KRAS |  |  |  |
| If IMRT + Cisplatin: |  |  |  |
| Non-variant | Reference | Reference | Reference |
| KRAS-variant | 0.48 (0.11, 2.06) | 0.45 (0.10, 1.90) | 0.48 (0.11, 2.03) |
| If IMRT + Cetuximab: |  |  |  |
| Non-variant | Reference | Reference | Reference |
| KRAS-variant | 1.50 (0.64, 3.51) | 1.51 (0.64, 3.58) | 1.38 (0.59, 3.23) |
|  | | | |
| Assigned treatment |  |  |  |
| If Non-variant: |  |  |  |
| IMRT + Cisplatin | Reference | Reference | Reference |
| IMRT + Cetuximab | 1.03 (0.57, 1.86) | 1.10 (0.61, 2.00) | 1.05 (0.58, 1.90) |
| If KRAS-variant: |  |  |  |
| IMRT + Cisplatin | Reference | Reference | Reference |
| IMRT + Cetuximab | 3.19 (0.66, 15.34) | 3.74 (0.77, 18.19) | 3.04 (0.63, 14.66) |
|  | | | |
| Age (years) |  | 0.4884 |  |
| Continuous, per 1-year increment |  | 1.013 (0.977, 1.050) |  |
|  | | | |
| Gender |  | 0.1727 |  |
| Female |  | Reference |  |
| Male |  | 2.69 (0.65, 11.13) |  |
|  | | | |
| Zubrod performance status |  | 0.3035 |  |
| 0 |  | Reference |  |
| 1 |  | 1.37 (0.75, 2.47) |  |
|  | | | |
| Smoking history (pack-years) |  | 0.9047 |  |
| Continuous, per 1-year increment |  | 1.001 (0.989, 1.013) |  |
|  | | | |
| T stage (AJCC 7th edition) |  | 0.1521 |  |
| T2-T4 |  | 1.80 (0.80, 4.04) |  |
|  | | | |
| N stage (AJCC 7th edition) |  | 0.0131 | 0.0074 |
| N0-N2b |  | Reference | Reference |
| N2c-N3 |  | 2.06 (1.16, 3.66) | 2.16 (1.23, 3.80) |
|  | | | |
| RTOG 0129 risk group* |  | 0.9090 |  |
| Low |  | Reference |  |
| Intermediate |  | 1.04 (0.50, 2.17) |  |
|  | | | |
| Bayesian Information Criterion (BIC) | 658.085 | 671.530 | 655.505 |
|  | | | |
| HR, hazard ratio; CI, confidence interval; AJCC, American Joint Committee on Cancer. *Low: >10 pack-years and N0-N2a, or ≤10 pack-years; intermediate: >10 pack-years and N2b-N3. | | | |
