# Supplementary material for: The KRAS-Variant and Cetuximab in HPV-Positive Oropharyngeal Cancer: Results from the NRG/RTOG 1016 Trial
Source: Cancer Res Commun. 2026 Mar 31;6(3):706–13. doi: 10.1158/2767-9764.CRC-25-0551 (PMC13036839; doi:10.1158/2767-9764.CRC-25-0551)
Supplement: Supplementary Table 11 — Grade 3-4 Treatment-Related [1] Skin Reaction Inside Portal [2] by KRAS and Assigned Treatment [file crc-25-0551_supplementary_table_11_suppst11.docx]

| **Supplemental Table 11: Grade 3-4 Treatment-Related [1] Skin Reaction Inside Portal [2] by KRAS and Assigned Treatment** | | | | |
| --- | --- | --- | --- | --- |
| KRAS | Assigned Treatment | Patients | Events | Odds Ratio  (95% Confidence Interval) |
|  |  |  |  |  |
| KRAS-variant | IMRT + Cisplatin | 44 | 4 (9.1%) | Reference |
|  | IMRT + Cetuximab | 48 | 6 (12.5%) | 1.43 (0.38, 5.44) |
|  |  |  |  |  |
| Non-variant | IMRT + Cisplatin | 230 | 17 (7.4%) | Reference |
|  | IMRT + Cetuximab | 237 | 35 (14.8%) | 2.17 (1.18, 4.00) |
|  |  |  |  |  |
|  |  |  |  |  |
| Total |  | 559 | 62 (11.1%) | interaction p=0.5769 |
|  |  |  |  |  |
| Odds ratios estimated from logistic regression model with covariates KRAS (KRAS-variant vs. non-variant), treatment (IMRT + Cetuximab vs. IMRT + Cisplatin) and the interaction of KRAS and treatment.  [1] Definitely, probably, or possibly related to protocol treatment.  [2] CTCAE version 4 terms: dermatitis radiation; radiation recall reaction (dermatologic). | | | | |
