# Supplementary material for: The KRAS-Variant and Cetuximab in HPV-Positive Oropharyngeal Cancer: Results from the NRG/RTOG 1016 Trial
Source: Cancer Res Commun. 2026 Mar 31;6(3):706–13. doi: 10.1158/2767-9764.CRC-25-0551 (PMC13036839; doi:10.1158/2767-9764.CRC-25-0551)
Supplement: Supplementary Table 12 — Grade 3-4 Treatment-Related [1] Skin Reaction Outside Portal [2] by KRAS and Assigned Treatment [file crc-25-0551_supplementary_table_12_suppst12.docx]

| **Supplemental Table 12: Grade 3-4 Treatment-Related [1] Skin Reaction Outside Portal [2] by KRAS and Assigned Treatment** | | | | |
| --- | --- | --- | --- | --- |
| KRAS | Assigned Treatment | Patients | Events | Odds Ratio  (95% Confidence Interval) |
|  |  |  |  |  |
| KRAS-variant | IMRT + Cisplatin | 44 | 0 | Reference |
|  | IMRT + Cetuximab | 48 | 8 (16.7%) | Can not be estimated |
|  |  |  |  |  |
| Non-variant | IMRT + Cisplatin | 230 | 0 | Reference |
|  | IMRT + Cetuximab | 237 | 27 (11.4%) | Can not be estimated |
|  |  |  |  |  |
|  |  |  |  |  |
| Total |  | 559 | 35 (6.3%) |  |
|  |  |  |  |  |
| Odds ratios estimated from logistic regression model with covariates KRAS (KRAS-variant vs. non-variant), treatment (IMRT + Cetuximab vs. IMRT + Cisplatin) and the interaction of KRAS and treatment.  [1] Definitely, probably, or possibly related to protocol treatment.  [2] CTCAE version 4 terms: nail loss; pruritus; rash acneiform; rash maculo-papular. | | | | |
