# Supplementary material for: The KRAS-Variant and Cetuximab in HPV-Positive Oropharyngeal Cancer: Results from the NRG/RTOG 1016 Trial
Source: Cancer Res Commun. 2026 Mar 31;6(3):706–13. doi: 10.1158/2767-9764.CRC-25-0551 (PMC13036839; doi:10.1158/2767-9764.CRC-25-0551)
Supplement: Supplementary Table 13 — Representativeness of Study Participants [file crc-25-0551_supplementary_table_13_suppst13.docx]

| **Supplementary Table 13.** Representativeness of Study Participants | |
| --- | --- |
| Cancer type(s)/subtype(s)/stage(s)/condition | HPV-positive oropharyngeal cancer (OPC), Head and Neck Cancer (HNSCC) |
| Considerations related to: | |
| Sex | General ratios are male:female as 4:1. |
| Age | Current trends in incidence for HPV+ OPC are 57 to 64. |
| Race/ethnicity | HPV+ OPC is most common in white males. |
| Geography | Most common in Western, high-income regions, especially North America |
| Other considerations | Trial accrual is often less common in minorities. Underrepresentation in trials limits evaluation of the impact of racial/ethnic- or ancestry-based differences in efficacy and toxicity. |
| Overall representativeness of this study | The age distribution of our study of 59 is similar to the average age distribution. The study population was from North America, where this disease is common.  This study had a higher predominance of male patients than female considering the normal incidence. This study was predominantly white (93%), which is higher than the normal distribution. These findings likely reflecting trial accrual trends.  The study reflected the accrual patterns to trials commonly seen, yet is not fully representative of US based HPV+ OPC HNSCC populations. |
